# Population genomics of heterosporous lycophytes: allopatric speciation and historical persistence through paleoclimatic fluctuations

**DOI:** 10.1111/tpj.71179

**Published:** 2026-11-04

**Authors:** TongXin Ye, Ran Wei, JiGao Yu, NingXin Wang, SiQi Liang, XianChun Zhang

**Affiliations:** ^1^ State Key Laboratory of Plant Diversity and Specialty Crops, Institute of Botany Chinese Academy of Sciences Beijing 100093 China; ^2^ China National Botanical Garden Beijing 100093 China; ^3^ University of Chinese Academy of Sciences Beijing 101408 China

**Keywords:** demographic history, geographical isolation, natural selection, Selaginellaceae, *Selaginella sanguinolenta* group

## Abstract

Speciation is the fundamental process driving the origin of earth biodiversity. While speciation mechanisms have been revealed in many plant taxa, the evolutionary dynamics of heterosporous lycophytes remain largely unexplored. Here, we used the *Selaginella sanguinolenta* group, comprising seven montane species in eastern Asia, as a model system. We generated a high‐quality, chromosome‐level reference genome for *S*. *aitchisonii*, conducted whole‐genome resequencing of 191 individuals across 49 populations, and integrated population genomic, phylogenomic, morphological, and ecological analyses. Our results revealed that strong geographic isolation, driven by Miocene paleogeographic events, led to high genetic differentiation and restricted interspecific gene flow. The unique combination of heterospory and independent gametophyte generations might exacerbate extreme inbreeding, further impeding the ability of these lineages to surmount geographical barriers. Ecological niche divergence indicated that these species experience heterogeneous selection pressures; moreover, evidence of selective sweeps, leaf arrangement variation, and functional gene divergence indicates that natural selection gives rise to new species with environmentally adaptive traits. Demographic reconstructions revealed a shared trajectory of expansion followed by contraction into bottlenecks from the late Miocene to the Pliocene. Montane refugia likely played a role in species persistence, preserving genetic diversity for the postglacial expansions observed in four species since the Last Interglacial. We conclude that the *S. sanguinolenta* group underwent allopatric speciation since the Miocene and successfully persisted through severe Quaternary climatic fluctuations, which provides novel insights based on population genomic analysis into the evolution of early‐diverging vascular plants.

## Summary

Speciation studies of heterosporous lycophytes under a population genomic framework are currently lacking. Using the *Selaginella sanguinolenta* group, we reveal how paleogeographic isolation, compounded by heterospory‐facilitated inbreeding, drove allopatric speciation since the Miocene, followed by subsequent ecological adaptation. These findings elucidate the evolutionary trajectory of ancient vascular plants, highlighting their resilience to Quaternary climatic oscillations and filling a critical gap in plant biodiversity research.

## INTRODUCTION

Understanding the mechanisms driving speciation—the fundamental process underlying the emergence and maintenance of great biodiversity on Earth—has remained a central goal in evolutionary biology for centuries (Barton, 2010; Cutter, 2023; Darwin, 1859; Wang & Liu, 2025). Recently, advancements in large‐scale genome sequencing have enabled researchers to explore this longstanding question across diverse plant lineages, for example, mangroves in angiosperms (He et al., 2019), *Pinus* in gymnosperms (Ren et al., 2012), *Gymnosphaera* in ferns (Wang et al., 2020), and *Sphagnum* in bryophytes (Piatkowski et al., 2023). However, lycophytes, as the earliest terrestrial vascular plants and the sister group to euphyllophytes (including ferns and seed plants) (Kenrick & Crane, 1997), remain conspicuously understudied in this context. Meanwhile, among the more than 1300 recognized lycophytes species (PPG I, 2016), over 70% are heterosporous, producing both megaspores and microspores. This lineage represents the oldest (originated in the Late Devonian) of three heterosporous lineages (*Selaginella* and *Isoetes* in lycophytes, the water ferns, seed plants), possessing a trait widely regarded as a crucial innovation for land plants (Bateman & DiMichele, 1994; Niklas & Kutschera, 2009; Petersen & Burd, 2017). Notably, unlike seed plants whose gametophytes entirely depend on the sporophyte, heterosporous lycophytes retain an independent gametophyte generation. This unique reproductive biology fundamentally distinguishes them from homosporous ferns (e.g., *Asplenium*, De Groot et al., 2012; *Athyrium*, Liang et al., 2022) and also differs from seed plants in which gametophyte entirely relies on the sporophyte. Despite their profound evolutionary significance, the specific mechanisms driving speciation within this remarkable group, as well as their strategies for persisting to the present day, remain largely unresolved.

Geographic isolation is widely considered a primary driver of speciation, as physical barriers to gene flow allow populations to accumulate genetic differentiation via mutation, natural selection, or genetic drift (Kulmuni et al., 2020; Stankowski & Ravinet, 2021). According to modern research on the mechanisms of speciation, the degree of geographic isolation between species is often regarded as an essential prerequisite for understanding how new species arise. Along the gradient of isolation, three principal scenarios are recognized: allopatric, parapatric, and sympatric speciation (Butlin et al., 2008; Coyne & Orr, 2004; Savolainen et al., 2006). Among these, allopatric speciation is the most frequently investigated (Mayr, 1963; Rieseberg, 2019), which is often a consequence of crustal movement (e.g., plate collision, volcano activity) (Chatterjee & Scotese, 2010) or glaciation (Wallis et al., 2016), and is particularly prevalent among plant lineages distributed across mountainous landscapes (Gorospe et al., 2025; Iseli et al., 2023; Ma et al., 2021). Nevertheless, incomplete geographic isolation does not necessarily indicate a weakening of the speciation process (Olivares et al., 2025). Current evidence reports that hybridization and speciation with gene flow can also play crucial roles in the formation of new species and the generation of biodiversity, especially in the taxa with overlapping distributions (sympatric distribution) (Feder et al., 2012; Li et al., 2021; Wang, Wu, et al., 2022; Wu et al., 2022), or those utilizing long‐distance seed dispersal or wind‐pollination mechanisms (Adams et al., 2020). Since geographic isolation has not been a focus in traditional lycophyte studies, and spore dispersal is more commonly examined in colonization‐related research (e.g., Troia, 2016), there is a limited understanding of its role in lycophyte speciation, especially how heterospory with independent generations influences geographic isolation and gene flow.

Since the publication of *On the Origin of Species* (Darwin, 1859), natural selection has been recognized as a central process in evolution. Generally, divergent natural selection drives the accumulation of genetic and phenotypic trait variations between populations (Via, 2009). These variations increase adaptation and reinforce barriers to gene flow, and reproductive isolation may result as a by‐product (Via, 2009). Recent theories of ecological selection emphasize the divergent selection which is driven by habitat differences, can directly promote genomic differentiation, and restrict gene flow, ultimately shaping new species even without the strict need for geographical barriers (Feder et al., 2012; Ke et al., 2022; Nosil et al., 2009). On the contrary, the specific trajectories of natural selection are frequently and strongly correlated with the emergence of distinct phenotypes. In lycophyte species, existing studies have identified several morphological and physiological innovations that facilitated adaptation to extreme environments, notable examples including the complex gene regulatory networks for desiccation tolerance in resurrection *Selaginella tamariscina* (Xu et al., 2018) and cup‐shaped bizonoplasts in *S. erythropus* for adaptation to low‐light habitat (Sheue et al., 2007). Nevertheless, the link between morphology or genetic mechanisms and the environment is rarely this intuitive; natural selection pressure is complex and subtle, especially among morphologically similar, closely related species. At least for lycophytes, the issue of how natural selection contributes to more widespread cases of speciation remains unexplored.

Furthermore, speciation is not an evolutionary endpoint. Most extant plant lineages diverged before or during the early Quaternary, indicating their long histories (Benton et al., 2022; Kadereit & Abbott, 2021). Since their initial divergence, plant species may have been continuously shaped by profound paleoenvironmental events, including dramatic climate fluctuations (Yin et al., 2021), shifting biotic interactions such as animal‐mediated dispersal or predation (Lorenz, 2008), and even mass extinction (Berry, 2021). Consequently, their population size, geographic distributions, and genetic structure have likely changed repeatedly through expansion and contraction (Griffiths et al., 2019), bottlenecks (Ony et al., 2021), and migration (Vitasse et al., 2021), often acting in concert (Fan et al., 2018). Quaternary climate oscillations, in particular, have been shown to drive widespread range contractions and retreats to refugia (areas of long‐term climatic stability) that served as reservoirs of biodiversity and facilitated postglacial recolonization (Provan & Bennett, 2008). Remarkably, lycophytes have persisted through mass extinction events across geological time. Unraveling their post‐speciation survival may offer valuable insights into the mechanism of long‐term lineage evolution, ultimately informing conservation and management efforts in the face of ongoing global climate change.

In this study, we particularly focus on the *Selaginella sanguinolenta* group as representative taxa, belonging to the most diverse family Selaginellaceae in heterosporous lycophytes. Based on previous studies, this species group is presumed to be the earliest tracheophyte lineage of allopolyploid origin (early Triassic), which underwent long‐term diploidization evolution, with the crown group divergence in the Miocene (Kang et al., 2024). The latest species delimitation study (Zhang et al., 2023) identified seven species within this group, comprising two widely distributed: *S. aitchisonii*, *S. jacquemontii*; and five narrowly distributed species: *S. baodongii*, *S. nummularifolia*, *S. rossii*, *S. sajanensis*, *S. sanguinolenta*, all of which are found across mountainous regions with diverse ecological conditions, from the Himalayas to Siberia (Figure 1). Taken together, they provide an outstanding background for studying speciation across multiple species with a complex origin, wide distribution, varied habitats, and long‐term evolution. Here, we propose three specific questions about the *S. sanguinolenta* group as insights into the lycophyte speciation mechanism with demographic history: (i) What roles do geographic isolation and natural selection play in their speciation? (ii) What demographic events have they gone through since speciation? (iii) Do heterospory or independent gametophyte generations have a special influence on the process of species evolution and development? To investigate these questions, we utilize a chromosome‐level genome assembly of *S. aitchisonii* as a reference for a series of commonly effective population genomic analyses. By integrating molecular evidence with ecological, morphological, and geographical data, this study aims to address the current knowledge gap in speciation in early heterosporous vascular plants and underscore their potential in broader plant evolutionary research.

**Figure 1:**
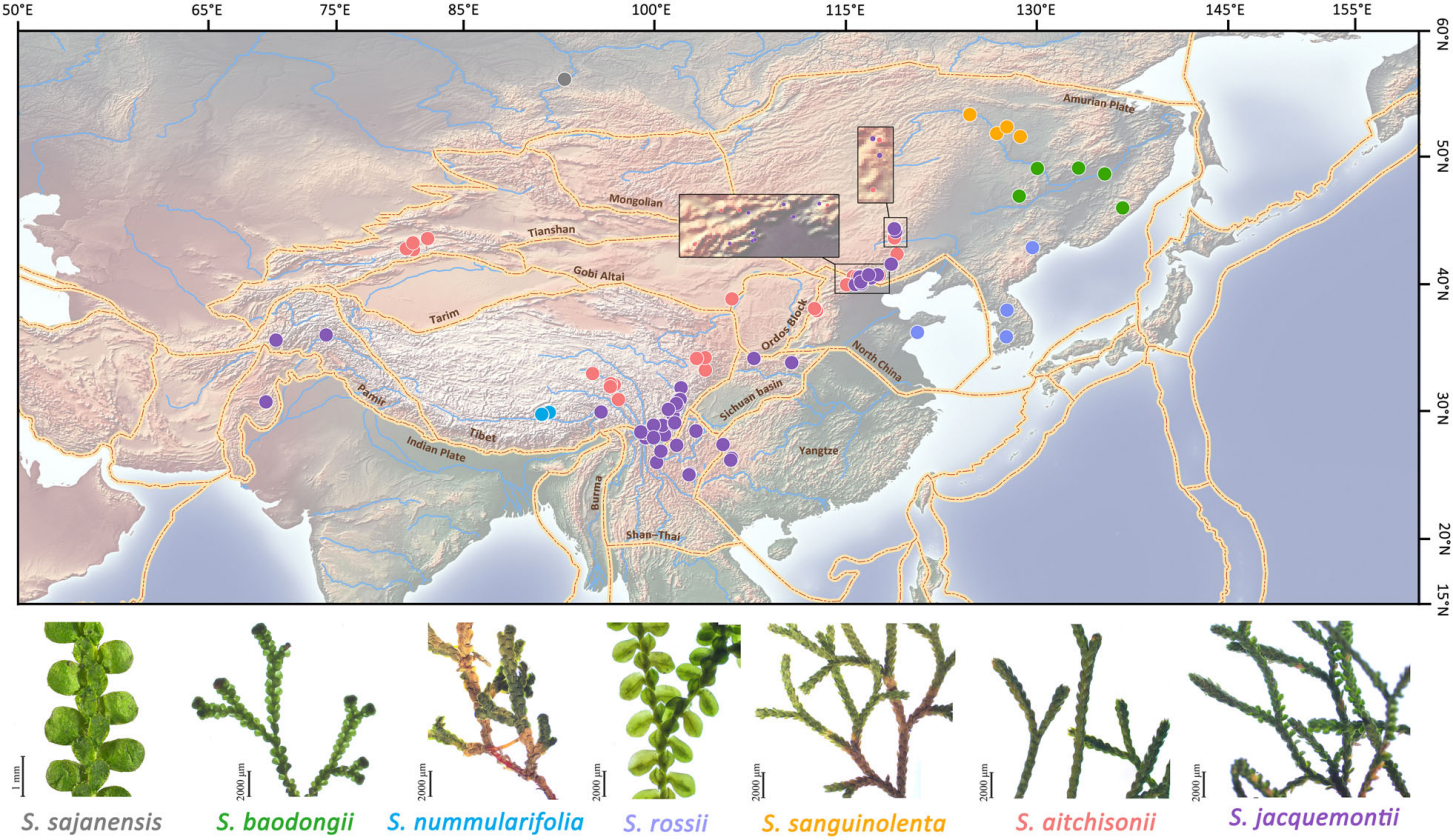
Distribution and morphology of seven species in the *Selaginella sanguinolenta* group. The color of sampling locations of each species marked on the map (dots) matches that of the species name below the images. Brown dotted lines with a yellow background represent tectonic plate boundaries from Hasterok et al. (2022). (Photograph of *S. sajanensis* from Zhang et al., 2023; others by Ye TX).

## RESULTS

### Genome assembly

To assemble the chromosome‐level genome of *Selaginella aitchisonii* as a reference for subsequent population genomic analyses, we initially generated a genome size of 197.58 Mbp based on a genome survey by K‐mer 17, showing the heterozygosity (0.84%) and repetitive (59.89%) sequences, respectively (Figure 2a; Tables S1 and S2). Afterwards, we obtained a draft assembled genome of 337.08 Mbp with a contig N50 value reaching 3.17 Mbp, containing 914 contigs (Table S3). Although the preliminary assembly results of the genome size are much larger than the survey, we did not observe significant redundancy peaks (Figure 2b). We successfully removed redundancy through automatic correction of HIC data (Figure 2c), resulting in the final genome size of 134.83 Mbp (anchored on 10 pseudochromosomes; Table S4) (Skaptsov et al., 2020).

**Figure 2:**
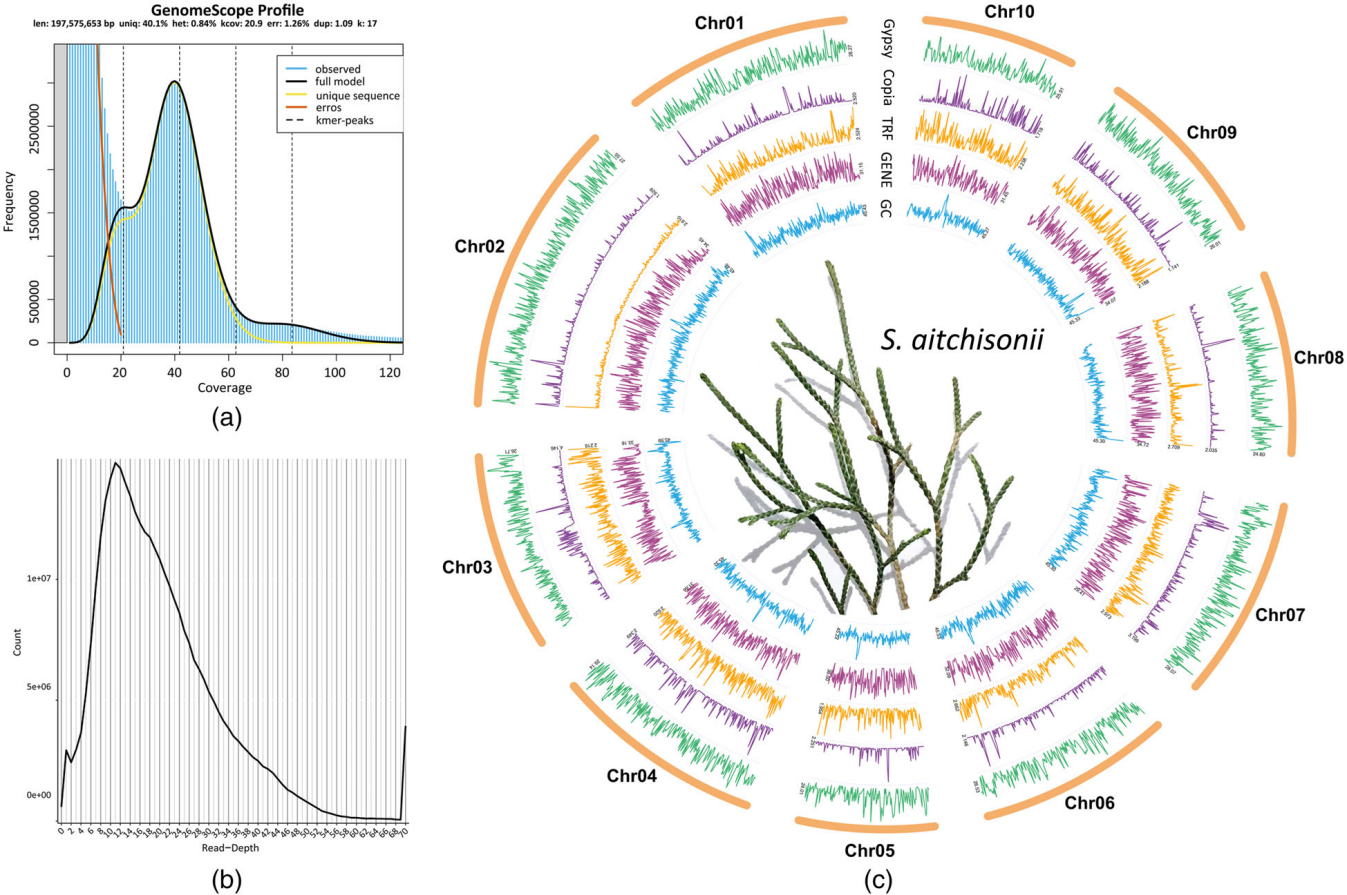
Genome features of *Selaginella aitchisonii*. (a) Genome survey of *S. aitchisonii* based on 17‐mer frequency distribution of *S. aitchisonii* plotted by GenomeScope. The x‐axis represents k‐mer depth, and the y‐axis represents k‐mer frequency. (b) Read depth distribution map of the preliminary assembly of *S. aitchisonii* generated by purge_haplotigs. (c) Circular representation of the *S. aitchisonii* genome. From outermost to innermost: chromosome length (scale in Mb), Gypsy LTR retrotransposon density, Copia LTR retrotransposon density, tandem repeat frequency (TRF) density, gene density, and GC content, all calculated in 50‐kb sliding windows.

Multiple strategies were used to assess the quality and completeness of the *S. aitchisonii* genome. First of all, 97.55% of PacBio HiFi reads were mapped to the 10 pseudochromosomes (Table S4), dispelling concerns about genome assembly integrity. Then, Benchmarking Universal Single‐Copy (BUSCO) analysis showed 89% genes of the embryophyte for the *S. aitchisonii* assembly genome (Table S4). We also used the LTR Assembly Index (LAI) to evaluate the assembly quality, and a high LAI value of 18 indicated that the *S. aitchisonii* genome reached the reference level (Table S5). Additionally, the *S. aitchisonii* genome contains 47.3% annotated repetitive sequences, of which LTR retrotransposons account for 69.57%, and Gypsy elements make up 83.24% of the LTRs (Table S5). After masking repeat sequences, we predicted 26 089 genes, of which 22 588 genes had functional annotation (Table S5).

### Whole‐genome resequencing and SNP detection

We generated a total of 1171.8 gigabases (Gb) of raw whole‐genome resequencing data from 191 individuals across the seven species (Figure 1), with 7 995 566 217 reads, with an average 41 861 603 reads per individual. After aligning with the reference genome, we obtained massive SNPs of each individual; and number of raw SNPs, mapping rate, coverage, and mean depth were recorded in Table S6. By restricting filter conditions from the GATK v4.4, we finally obtained 18 633 942 high‐quality SNPs before the rigorous filtering, the detailed SNPs quantities after vary filtering parameters (e.g., missing rates) were recorded in Tables S7 and S8.

### Population structure

We used the unrooted Neighbor‐Joining (NJ) tree, ADMIXTURE (Alexander et al., 2009), and principal component analysis (PCA) to explore the patterns of genetic structure of the *Selaginella sanguinolenta* group (Figure 3, Figures S1 and S2). Based on the NJ tree (Figure 3a), seven monophyletic species cluster into three main clades. Clade I contained five species with narrow wild distribution (*S. baodongii*, *S. nummularifolia*, *S. rossii*, *S. sajanensis*, *S. sanguinolenta*), whereas widely distributed species *S. aitchisonii and S. jacquemontii* formed Clade II and Clade III, respectively. ADMIXTURE analyses give similar results (Figure 2b). Across all individuals, the CV error (Figure S1a) decreased continuously from *K* = 1 to *K* = 10, with the decline plateauing after *K* = 5. At *K* =  2, Clade II (*S. aitchisonii*) and Clade III (*S. jacquemontii*) were categorized into a distinct genetic cluster. For Clade I, the ADMIXTURE results (extracted from the whole dataset) showed clear population structure that matched with species (Figure 3b). Both mono‐species Clade II (*S. aitchisonii*) and Clade III (*S. jacquemontii*) showed intraspecies structure (Figure 3b) which were strongly related to geographical distribution. *S. aitchisonii* in Xinjiang populations formed a pure cluster. Three populations from Qinghai and Xizang were clustered into a single branch. The populations distributed in northern China, including Nei Mongol, Beijing, Hebei, and Shanxi, were closely related and clustered into a large branch. Two geographically close populations from southern Gansu and northern Sichuan form a mixture cluster. Clade III (*S. jacquemontii*) showed clear north–south differentiation (Figure 3b). The Hengduan Mountains lineage consists of Sichuan, Yunnan, and Guizhou populations. *S. jacquemontii* growing in northwest to southwest China seemed more complicated. The individuals from Beijing and Nei Mongol were scattered on different NJ tree branches and genetic clusters (Figure 2a). PCA analysis (Figure S2) were basically consistent with those of NJ tree and ADMIXTURE. The analysis of molecular variance (AMOVA) showed *S. sanguinolenta* group explained 30.82% of the genetic variance across species, compared to 10.74% among populations within species and 58.43% within populations (*P* < 0.05, Table S9).

**Figure 3:**
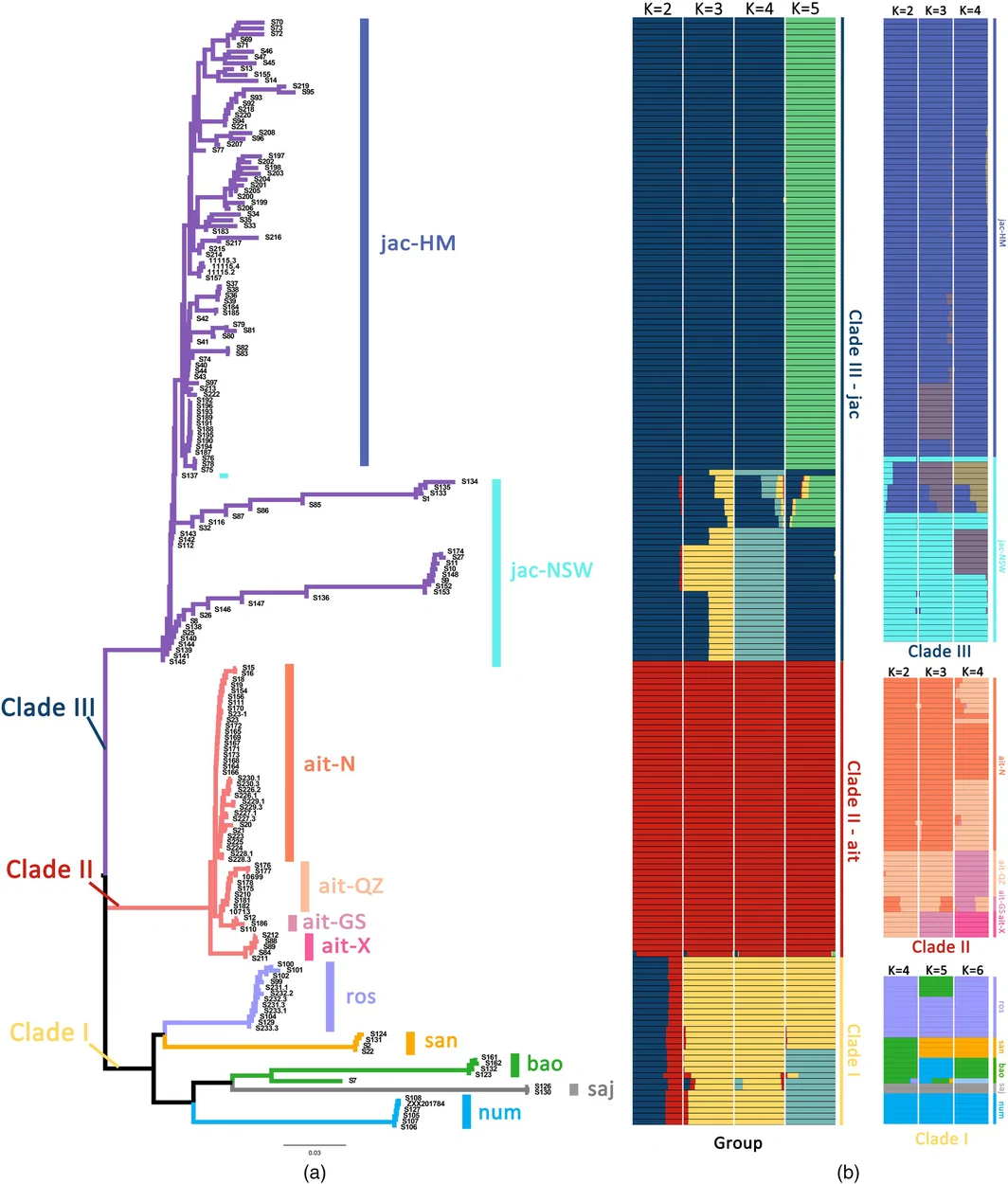
Population structure of seven species in the *Selaginella sanguinolenta* group. (a) Neighbor‐Joining tree, (b) results of ADMIXTURE analyses. *ait‐*S. aitchisonii*, jac‐*S. jacquemontii*, bao‐*S. baodongii*, num‐*S. nummularifolia*, ros‐*S. rossii*, saj‐*S. sajanensis*, san‐*S. sanguinolenta*; X‐Xiangjiang, QZ‐Qinghai and Xizang, N‐northern China, GS‐southern Gansu and northern Sichuan, HM‐Mt. Hengduan, NSW‐northwest and southwest China.

### Genetic diversity and differentiation

To mitigate the impact of linkage disequilibrium (LD) on sliding window analysis, we first estimated LD decay based on the coefficient of determination (*r* ^2^) using PopLDdecay (Zhang, Dong, et al., 2019). The analysis indicated that LD decay reached a lower level over the distance of 10 kb (Figure S3). Thus, in the following sliding window analysis, non‐overlapping 10 000 bp windows with 10 000 bp steps were used. To explore the genetic diversity across three clades with seven species, the genetic parameters, containing nucleotide diversity (*π*), observed (*H*_o_), expected (*H*_e_) heterozygosity, and inbreeding coefficient (*F*) were calculated, respectively (Table 1). The results suggested that the genetic diversity of the *Selaginella sanguinolenta* group was relatively low. The total group *π* was 0.014 ± 0.011, with the seven species ranging from 0.005 ± 0.008 (*S. sajanensis*) to 0.014 ± 0.008 (*S. jacquemontii*). Genetic heterozygosity is low in our species group, *H*_o_ and *H*_e_ ranging from 0.008 ± 0.0003 (*S. sajanensis*) to 0.085 ± 0.0509 (*S. jacquemontii*) and from 0.313 ± 0.0002 (*S. rossii*) to 0.32 ± 0.0002 (*S. aitchisonii*), respectively; the group *F* was 0.813 ± 0.157, ranging from 0.975 ± 0.0009 (*S. sajanensis*) to 0.732 ± 0.162 (*S. jacquemontii*). The species group showed a high level of genetic differentiation between species (Figure 4a), average fixation index (*F*_ST_) ranged from 0.47 (*S. aitchisonii* versus *S. sajanensis*) to 0.67 (*S. rossii* versus *S. sanguinolenta*) and average absolute nucleotide divergence *d*_XY_ ranged from 0.03 (*S. aitchisonii* versus *S. jacquemontii*) to 0.047 (*S. nummularifolia* versus *S. jacquemontii*). Genetic differentiation of lineages (Figure 4b,c) were generally lower than those of species, *F*_ST_ of *S. aitchisonii* lineages pairs ranged from 0.31 (southern Gansu and northern Sichuan versus Qinghai and Xizang) to 0.50 (northern China versus Xinjiang), *d*_XY_ ranged from 0.013 (southern Gansu and northern Sichuan versus northern China) to 0.018 (northern China versus Xinjiang); *F*_ST_ between *S. jacquemontii* lineages (Mt. Hengduan versus northwest and southwest China) is 0.31, *d*_XY_ is 0.011.

**Table 1:** Genetic diversity indices in different population levels: *π*‐Nucleotide diversity, *H*_o_‐Observed heterozygosity, *H*_e_‐Expected heterozygosity, *F*‐Inbreeding coefficient

Population	π	*H*_o_	*H*_e_	*F*
Group	0.014 ± 0.011	0.059 ± 0.049	0.318 ± 0.002	0.813 ± 0.157
Clade I	0.022 ± 0.012	0.060 ± 0.037	0.318 ± 0.002	0.812 ± 0.118
Clade II (*S. aitchisonii*)	0.013 ± 0.007	0.028 ± 0.0066	0.32 ± 0.0002	0.914 ± 0.021
Clade III (*S. jacquemontii*)	0.014 ± 0.008	0.085 ± 0.0509	0.317 ± 0.0013	0.732 ± 0.162
*S. sajanensis*	0.005 ± 0.008	0.008 ± 0.0003	0.315 ± 0.00002	0.975 ± 0.0009
*S. nummularifolia*	0.011 ± 0.011	0.015 ± 0.0008	0.315 ± 0.00014	0.954 ± 0.002
*S. rossii*	0.009 ± 0.008	0.023 ± 0.0021	0.313 ± 0.0002	0.928 ± 0.007
*S. sanguinolenta*	0.008 ± 0.008	0.013 ± 0.0007	0.316 ± 0.00003	0.959 ± 0.002
*S. baodongii*	0.013 ± 0.008	0.011 ± 0.0021	0.316 ± 0.0002	0.964 ± 0.007
*S. aitchisonii*‐GS	0.008 ± 0.007	0.043 ± 0.026	0.319 ± 0.001	0.864 ± 0.083
*S. aitchisonii*‐X	0.01 ± 0.008	0.042 ± 0.016	0.317 ± 0.003	0.866 ± 0.052
*S. aitchisonii*‐N	0.008 ± 0.006	0.057 ± 0.066	0.316 ± 0.002	0.821 ± 0.211
*S. aitchisonii*‐QZ	0.011 ± 0.008	0.19 ± 0.05	0.315 ± 0.001	0.918 ± 0.011
*S. jacquemontii*‐HM	0.002 ± 0.005	0.054 ± 0.032	0.318 ± 0.002	0.795 ± 0.101
*S. jacquemontii*‐NSW	0.01 ± 0.007	0.132 ± 0.05	0.316 ± 0.001	0.581 ± 0.168

X = Xiangjiang; QZ = Qinghai and Xizang; N = northern China; GS = southern Gansu and northern Sichuan; HM = Mt.Hengduan; NSW = northwest and southwest China.

**Figure 4:**
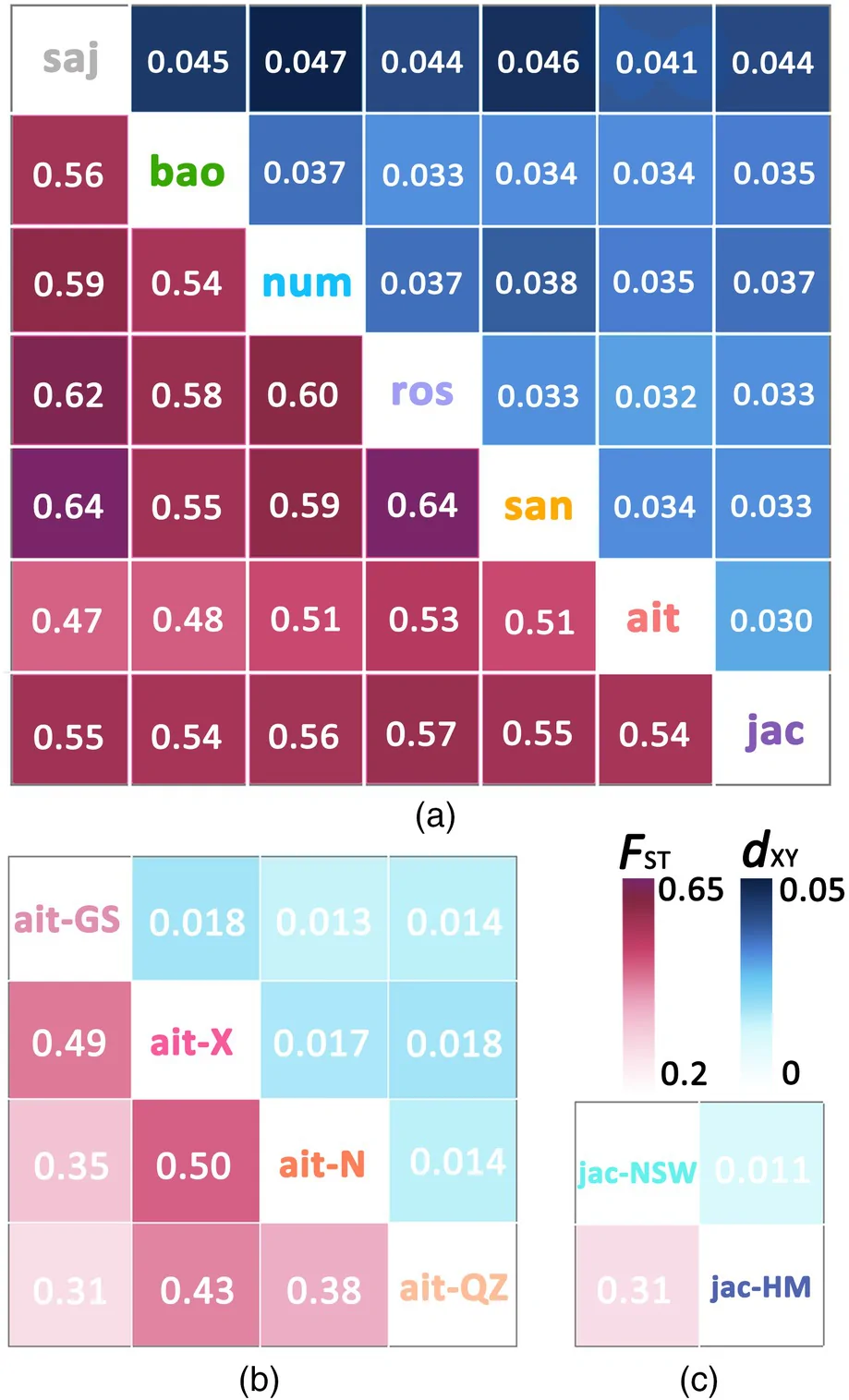
Genetic diversity and differentiation of seven species in the *Selaginella sanguinolenta* group. (a) Nucleotide diversity (*π*), average fixation index (*F*_ST_), and absolute nucleotide divergence (*d*_XY_) between species lineages of (b) *S. aitchisonii* and (c) *S. jacquemontii*, respectively, each in 10‐kb non‐overlapping genomic windows. *ait = *S. aitchisonii*; jac = *S. jacquemontii*; bao = *S. baodongii*; num = *S. nummularifolia*; ros = *S. rossii*; saj = *S. sajanensis*; san = *S. sanguinolenta*; X = Xiangjiang; QZ = Qinghai and Xizang; N = northern China; GS = southern Gansu and northern Sichuan; HM = Mt. Hengduan; NSW = northwest and southwest China.

### Phylogenetic relationships and gene flow

We reconstructed phylogenetic relationships among seven species and individuals (*S. sajanensis* as outgroup). The maximum likelihood (ML) tree using IQ‐TREE2 (Minh et al., 2020) showed excellent monophyly among species in the group (see the complete tree in Figure S4). The topology of a phylogenetic tree obtained by IQ‐TREE2 (SNPs concatenation maximum likelihood tree), TreeMix (populations allelic frequencies maximum likelihood tree) (Pickrell & Pritchard, 2012), and SVDquartets (SNPs coalescent species tree) (Chifman & Kubatko, 2014) was obviously inconsistent at the species level (Figure 5a–c). SVDquartets (Figure 5a) and TreeMix (Figure 5b, Figure S5) trees support a sister‐species relationship between *S. aitchisonii* and *S. jacquemontii* and between *S. rossii* and *S. sanguinolenta*. Rather than forming paired sister‐taxa clades, the IQ‐TREE tree (Figure 5c) resolves a sequential, stepwise divergence of lineages. *S. aitchisonii* represents the early‐diverging lineage, followed by the successive branching of *S. nummularifolia*, *S. baodongii*, *S. sanguinolenta*, and *S. rossii*, ultimately terminating in a highly divergent *S. jacquemontii* clade (see the complete tree in Figure S4). Based on SplitsTree (Huson & Bryant, 2006), all species have independent origins (Figure 5d). TWISST (Martin & Van Belleghem, 2017) analysis indicated topological inconsistency, as no one dominant topology emerged from the candidate structures (Figure S6).

**Figure 5:**
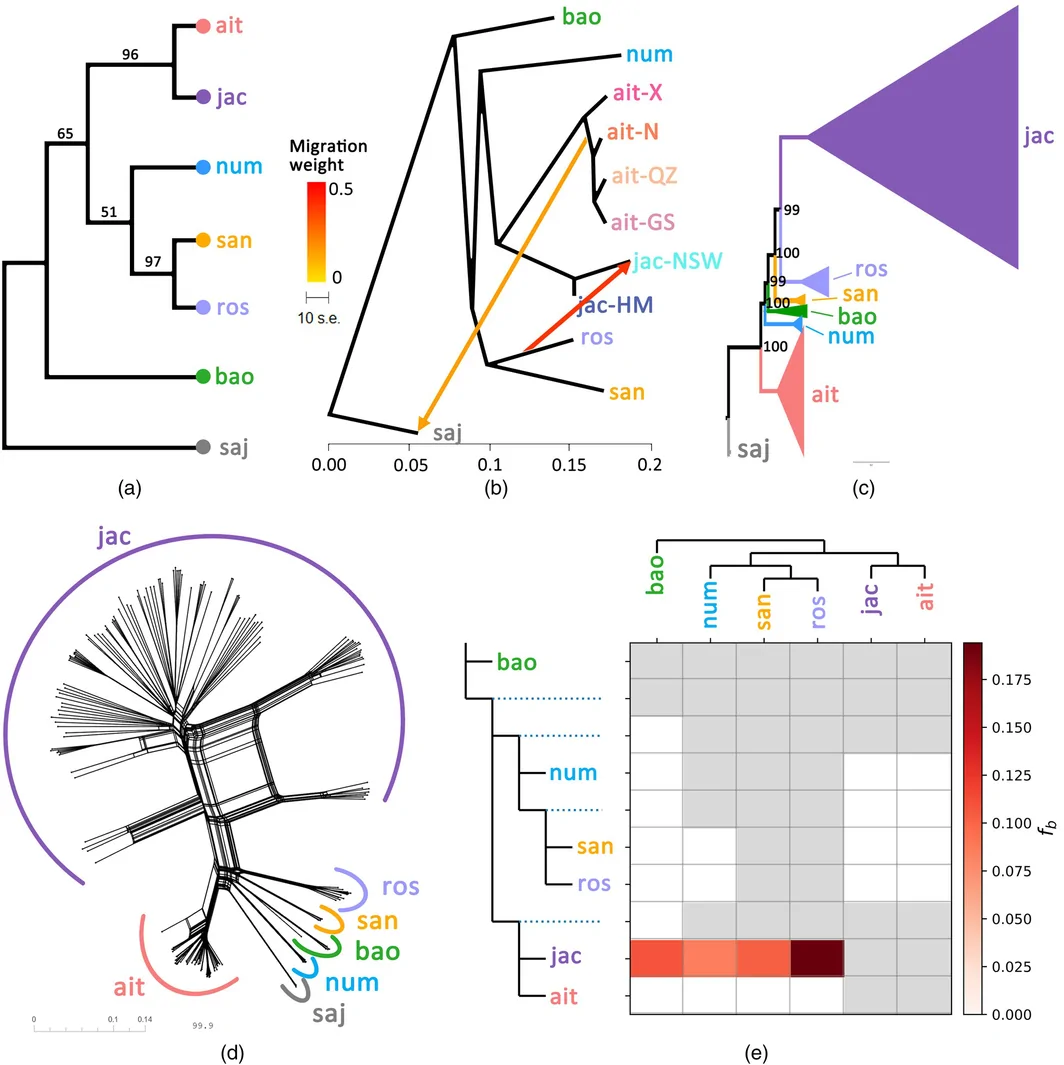
Phylogenetic relationships and gene flow of seven species in the *Selaginella sanguinolenta* group. (a) SVDQuartets SNPs coalescent species tree, (b) TreeMix allelic frequencies ML population tree under two (optimal) gene flow, (c) SNPs concatenation maximum likelihood (ML) individual tree, (d) events of gene flow based on *f*‐branch metric identified by Dsuite, and (e) phylogenetic network inferred with SplitsTree. *ait‐*S. aitchisonii*, jac‐*S. jacquemontii*, bao‐*S. baodongii*, num‐*S. nummularifolia*, ros‐*S. rossii*, saj‐*S. sajanensis*, san‐*S. sanguinolenta*; X‐Xiangjiang, QZ‐Qinghai and Xizang, N‐northern China, GS‐southern Gansu and northern Sichuan, HM‐Mt. Hengduan, NSW‐northwest and southwest China.

For interspecific introgression, the ABBA‐BABA tests by Dsuite (*S. sajanensis* as outgroup) (Malinsky et al., 2021) revealed seven significant weak signals among species. We defined gene flow between the species pair (i.e., P2 and P3 in the ABBA‐BABA analysis) with significant *D*‐value and *f*_4_ ratio (Table S10, Figure 5d). To refine these signals, we calculated *f*‐branch (*f*_b_) statistics, which allowed us to localize introgression events to specific phylogenetic branches. This analysis revealed that moderate historical introgression occurred related with the *S. jacquemontii* (involving *S. baodongii*, *S. nummularifolia*, *S. sanguinolenta*, and *S. rossii*). Further investigation using TreeMix to model historical splits and admixtures (Figure 5b; Figure S5) identified two optimal migration events (*Δ*m), explaining 99.8% of the variance, pinpointing gene flow from the common ancestor of *S. aitchisonii* lineages (northern China, southern Gansu and northern Sichuan, and Qinghai and Xizang) to *S. sajanensis*, as well as from *S. rossii* to *S. jacquemontii* lineage northwest and southwest China. SplitsTree network analysis revealed that this group has achieved distinct genetic divergence, but strong reticulation signals during early evolutionary stages or among closely related intraspecies lineages, indicating that traditional strictly bifurcating trees fail to accurately capture their topological relationships (Figure 5e). *N*_m_ (number of migrants per generation) gene flow values (formula: *N*_m_ ≈ (1‐*F*_ST_)/4*F*_ST_) exhibited extremely low level, ranging from 0.12 (*S. sajanensis* versus *S. sanguinolenta*) to 0.32 (*S. aitchisonii* versus *S. baodongii*) among species pairs (Figure S7).

### Morphological variation

To investigate the potential role of natural selection across these seven species, we examined their morphological traits and mainly focused the variation in vegetative leaf arrangement within the *Selaginella sanguinolenta* group (Figure 6a, Figure S8). Specifically, *S. rossii, S. sajanensis*, and *S. baodongii* possess typical dimorphic vegetative leaves arranged in four flat rows (Figure S8f,g,h). These species are characterized by smaller dorsal leaves and larger ventral leaves that alternate and stretch, partially exposing the stem. In *S. baodongii*, the size discrepancy between the dorsal and ventral leaves is less pronounced. Although *S. nummularifolia* also displays a four‐row flat leaf arrangement, it is distinguished by thick dorsal leaves that are notably larger than its ventral ones (Figure S8a). Conversely, the dorsal and ventral leaves of both *S. aitchisonii* and *S. sanguinolenta* exhibit minimal size differentiation and tend to closely clasp the stem (Figure S8b,c). *S. jacquemontii* features smaller dorsal leaves and larger ventral leaves, both clasping and flat arrangements can co‐occur on the same individual plant (Figure S8e,f). Furthermore, we found that these diverse structural arrangements can be categorized into three distinct cross‐sectional types, which we designated as: (i) fusiform (*S. nummularifolia*), (ii) rounded squared (*S. aitchisonii*, *S. sanguinolenta*, *S. jacquemontii*); (iii) horizontal H‐shape (*S. rossii,* *S. sajanensis, S. baodongii,* S. *jacquemontii*) (Figure 6a, Figure S8).

**Figure 6:**
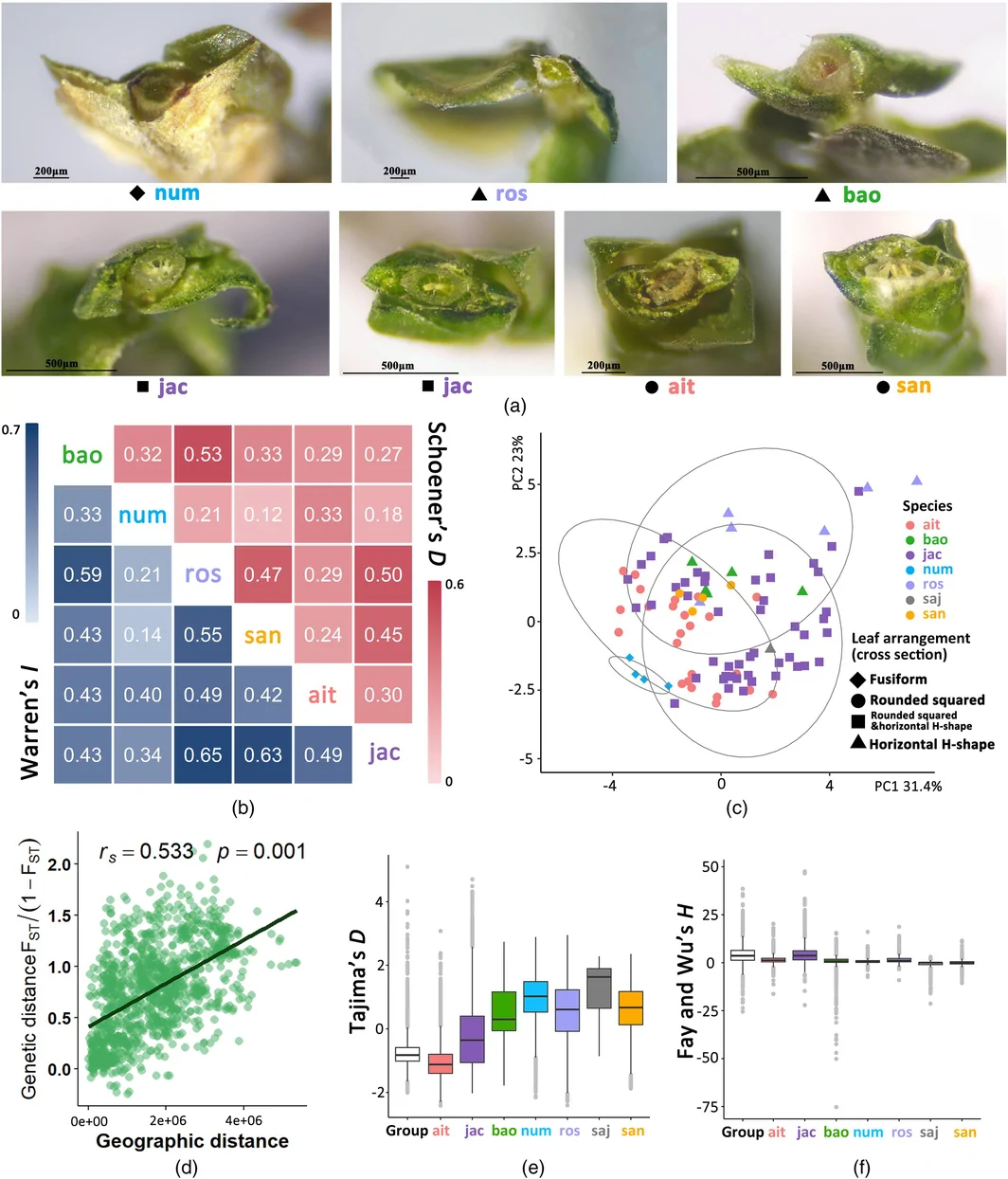
Natural selection assessment of seven species in the *Selaginella sanguinolenta* group. (a) Morphological observation of leave arrangement cross‐section, (b) Niche overlap statistics of Schoener's *D* and Warren's *I*, (c) principal component analysis of 18 environmental variables; different shapes represent the types of leave arrangement cross‐section. (d) Population isolation‐by‐distance, genetic distance using *F*_ST_/(1‐*F*_ST_) among populations. (e) Tajima's *D*, and (f) Fay and Wu's *H* of species, each in 10‐kb non‐overlapping genomic windows. *ait‐*S. aitchisonii*, jac‐*S. jacquemontii*, bao‐*S. baodongii*, num‐*S. nummularifolia*, ros‐*S. rossii*, saj‐*S. sajanensis*, san‐*S. sanguinolenta*.

### Ecological niche differentiation

To evaluate niche differentiation, we calculated niche overlap statistics using Schoener's *D* (0.12–0.53) and Warren's *I* (0.21–0.65) (Warren et al., 2010) (Figure 6b), which all indicated low to moderate niche overlap among species. Eighteen environmental factors' PCA were further applied (Table S11, Figure 6c). Subsequent analysis of variance (ANOVA) revealed significant differences across all evaluated environmental categories—temperature, precipitation, soil, solar radiation, wind speed, and topography—among the habitats of the seven species (Table S12). Within them, *S. nummularifolia* showed the strongest ecological separation, differing significantly across 13 factors. It occupied a distinct niche extreme, exhibiting maximum values for elevation, precipitation seasonality, solar radiation, soil total exchangeable bases, soil pH; alongside minimum values for temperature seasonality, max temperature of warmest month, annual precipitation, precipitation of wettest month, potential evapotranspiration, aridity index, and soil cation exchange capacity. Conversely, *S. rossii* occupied another extreme, mainly showing the maximum mean values in factors related to temperature and precipitation; the other five species were relatively intermediate (Table S12).

### Drivers of genomic differentiation and signatures of selection

Mantel tests revealed significant isolation‐by‐distance (IBD; *r*_*s*_ = 0.53, *P* < 0.001) (Figure 6d) and isolation‐by‐environment (IBE; *r*_*s*_ = 0.14, *P* < 0.001). Although the simple Mantel test initially suggested a weak correlation between genetic and environmental distances, environmental variables are frequently confounded by spatial autocorrelation (Wang & Bradburd, 2014). After strictly controlling geographic distance by the partial Mantel test (Legendre & Legendre, 2012), the IBE effect became completely non‐significant (*r*_*s*_ = 0.019, *P* = 0.400), revealing that the initial weak IBE signal was a statistical artifact driven by spatial autocorrelation. Tajima’ *D* statistics (Figure 6e) (Tajima, 1989) were negative (−0.721 ± 0.427) for the group, but not all the species showed significant departures from neutral equilibrium expectations, with the range from −1.058 ± 0.511 (*S. aitchisonii*) to 1.229 ± 0.892 (*S. sajanensis*); whereas Fay and Wu's *H* statistic (Figure 6f) (Fay & Wu, 2000) did not show significant positive selection. To further investigate how natural selection shapes genetic divergence, we analyzed highly differentiated genomic regions. Based on pairwise *Z*‐standardization *F*_ST_ values, we identified 269 highly differentiated genomic windows (top 2%) corresponding to 418 (*S. baodongii* versus *S. sajanensis*) to 950 (*S. aitchisonii* versus *S. rossii*) candidate genes (Table S13, Figure S9). In total, Gene Ontology (GO) functional enrichment analysis detected 1539 terms (*P‐value* <0.05), representing (305 non‐repetitive terms) from 12 (*S. aitchisonii* versus *S. baodongii*) to 135 (*S. aitchisonii* versus *S. nummularifolia*). After applying the Benjamini–Hochberg (BH) (Benjamini & Yekutieli, 2001), 221 terms (115 unique terms) remained significant (corrected *P*‐value <0.05). All identified GO terms and a summary of their counts are provided in Table S13 and Figure S9.

### Demographic history

Bayesian divergence time estimation with genome‐wide single‐nucleotide polymorphism (SNAPP) analysis (Stange et al., 2018) suggested that the divergence of seven species in the *Selaginella sanguinolenta* group occurred before the Pliocene (considering 95% highest probability density, HPD intervals) (Figure 7a). Afterwards, PSMC demographic inferences (Figure 7b) showed that the *N*_e_ of all seven species peaked between 4 and 3 mya followed by a drastic decline with bottleneck. Subsequently, the effective population size rebounded in four species (*S. aitchisonii*, *S. jacquemontii*, *S. nummularifolia*, *S. rossii*) during the late Pleistocene (0.7–0.2 mya), whereas the remaining three species retained a small and stable *N*_e_. Species distribution model (SDM) using MaxEnt (Phillips et al., 2006) (Figure 7c) showed that the simulated extent of highly suitable paleo‐habitats continuously decreased from the Marine Isotope Stage (MIS) M2 (~3.3 mya). By the end of the last glacial maximum (LGM), the optimum habitats declined to a fragmented state. Then, currently the area has recovered to a certain extent. Besides, the spatial pattern of population *π* showed that the girds with relatively higher values were located in the optimal habitat from 14.7 to 17 thousand years ago (kya) (Figure 7d), which mainly belonged to the distribution area of *S. aitchisonii* and *S. jacquemontii*.

**Figure 7:**
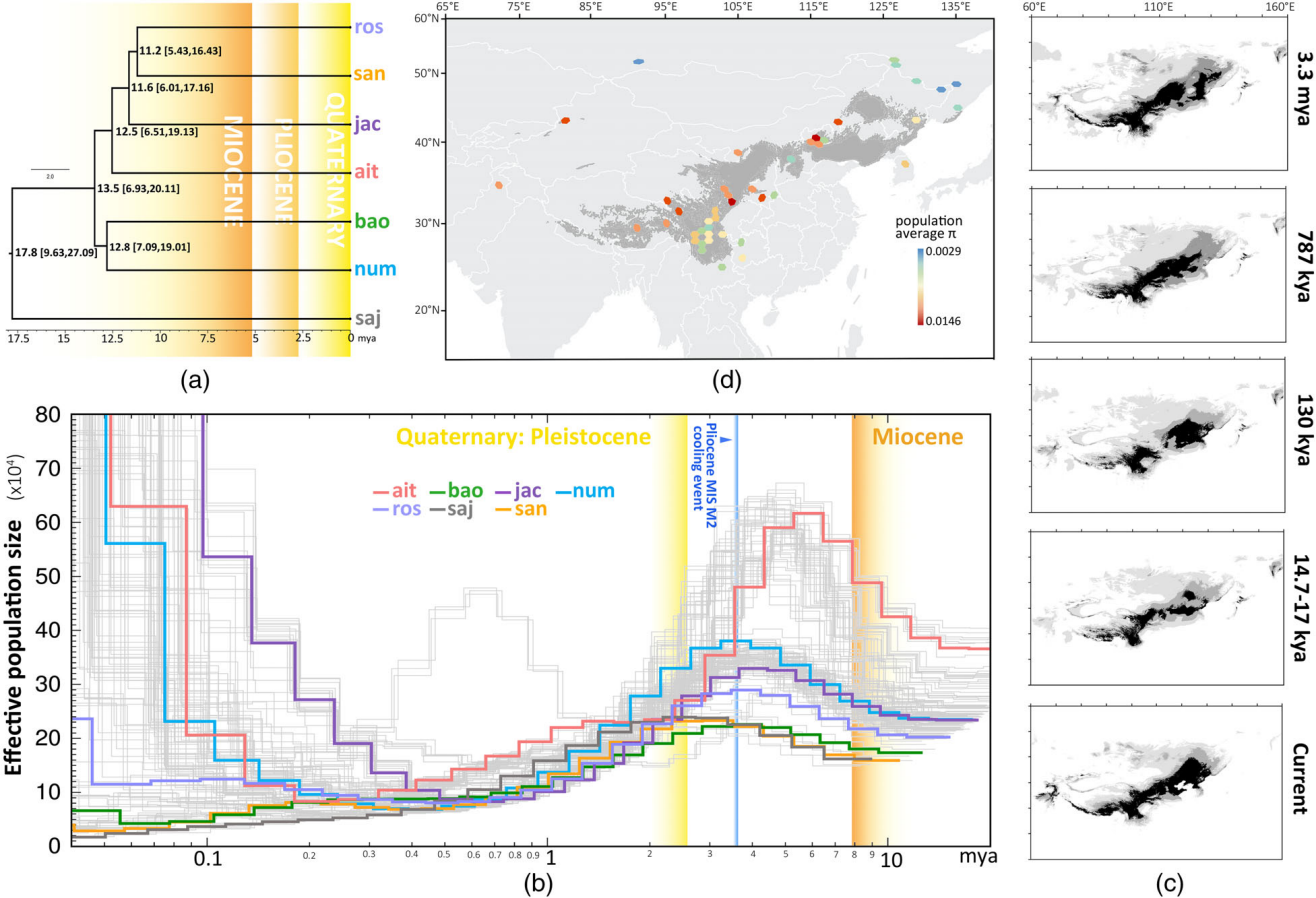
Demographic history of seven species in the *Selaginella sanguinolenta* group. (a) Species‐tree analysis with Newick‐formatted input and Phylogenetic‐tree prior (SNAPP) divergence time estimation. (b) Chronological variation of effective population size in seven species inferred by pairwise sequentially Markovian coalescence (PSMC) model, scaled using a generation time (g) of 5 years and a mutation rate (μ) of 0.7 × 10^−8^. (c) Results of ecological niche modeling of the group from paleo (3.3 mya, 787 kya, 130 kya, 14.7–17 kya) to current, and the areas in black represent the simulated optimal habitat. (d) Population nucleotide diversity (π) pattern with the map of optimal habitat in 14.7–17 kya.

## DISCUSSION

Based on our findings, seven species in the *Selaginella sanguinolenta* group may have undergone allopatric speciation initiated by geographic isolation since the Miocene, with natural selection acting subsequently to drive environmental adaptation. Following speciation, they experienced dramatic demographic fluctuations: expansion, contraction, bottlenecking, and several species undergoing a secondary expansion from the Late Miocene–Pliocene to the present. In the following modules, we comprehensively address our previous questions and provide outlooks for future studies.

### Speciation may start from geographic isolation caused by paleogeographic events

Based on literature and our field surveys, the current species in the *Selaginella sanguinolenta* group are typical mountainous plants in eastern Asia. Their populations exhibit a highly fragmented distribution, separated by steep peaks or deep canyons, with no sympatric populations or hybrid zones observed to date. Consistent with this geographical isolation, we found clear interspecies genetic structure (Figure 3) and high genetic differentiation (Figure 4a). Furthermore, the positive correlation in our IBD analysis (Figure 6d) demonstrates that geographic distance primarily drives this genetic divergence. Together, these findings support the classic model of allopatric speciation, where the geographic barriers facilitate reproductive isolation—a process similarly observed in the isolated population of Darwin's finches on Galápagos Island (Grant & Grant, 2009) and the divergence of electric fish caused by the closure of the Isthmus of Panama (Aguilar et al., 2019).

Afterwards, another question comes up: How do these specific isolations arise in the *Selaginella sanguinolenta* group? Unlike animals (e.g., *Tyrannus savanna*, Gómez‐Bahamón et al., 2020), we do not consider spikemosses to be organisms that are capable of movement, so that it can almost exclude active population migration as a reason. In addition, *S. sanguinolenta* group species divergences are not recent events, initiating from the late Oligocene to the early Miocene and completing in the early Pliocene (Figure 7a), a timeline that closely aligns with the recent estimates by Kang et al. (2024). It is well established that a series of major geological events, such as The Himalayan Collisional Orogeny, occurred in East Asia during the Cenozoic, greatly altering topography and atmospheric circulation (Wang, Kang, et al., 2022; Yu et al., 2022). Some scholars have pointed out that geographical and habitat isolation, caused by tectonic activity, intensified monsoons, and cycles of glaciation and deglaciation, may speed up in‐situ speciation (Pandit et al., 2025). Therefore, we believe it is essential to understand how these geographical events contribute to isolation, especially given the current distribution of species. The earliest drivers of speciation in this group were likely the neotectonic movements in the Sayan region, beginning during the Late Mesozoic to Early Cenozoic. The activation of tectonic movements in the Oligocene–Early Miocene resulted in ridges and depressions landscape, which are controlled by active faults (Arzhannikov et al., 2025), as the formation of possible extensive geographical barriers for *S. sajanensis*. The divergence of *S. nummularifolia* on the northern edge of the Eastern Himalayas and the Yarlung Zangbo River Basin may be related to the end of the Himalayan rapid uplift (around 23–19 mya) and the evolution of the Yarlung Zangbo River and the Irrawaddy River (Song et al., 2024). Meanwhile, during this period, denudation and erosion of the Himalayas caused by climate change may also led to geographic separation (Galy et al., 2010). In addition, the Himalayan orogeny may have also affected the main distribution areas of *S. aitchisonii* and *S. jacquemontii*, the second‐step terrain in China, for example, the uplift of Taihang–Lvliangshan region (Huang et al., 2022) and the Tianshan Mountains (Li et al., 2024). Since these two species eventually formed a distribution surrounding the Sichuan Basin, we speculate that their speciation would not have occurred earlier than the middle Miocene (ca. 13 mya) (Liu & Xu, 2003), which actually supports the results of our divergence time estimation. Additionally, in the eastern margin of the Eurasian (Amurian Plate), late Miocene to early Pliocene diverged into three species (*S. baodongii*, *S. rossii*, *S. sanguinolenta*) that maintained strong patterns of geographic isolation. Although the Amurian plate is rotating and moving eastward since the Pliocene (Heki et al., 1999), there is no evidence that any major mountain building took place in the region at that time. So, we noticed that the climate transition (from a period of cooling to a warmer period) at the Miocene–Pliocene boundary resulted in a rise in sea surface and changes in the water level of the Amur River basin, which may ultimately further block the land route for gene flow. Finally, through these sudden, disorderly and densely isolated events, seven closely related species formed in a genetic divergence pattern highly correlated with their geographical distribution.

### Natural selection drives environmental adaptation in new species

After allopatric differentiation restricts gene flow, natural selection and genetic drift become the two primary mechanisms driving changes in allele frequencies over time (Chen et al., 2019). Although recent theories propose that divergent ecological selection can directly promote speciation (Feder et al., 2012), our results rule out primary ecological speciation for the *Selaginella sanguinolenta* group. The absence of a significant IBE effect demonstrates that environmental heterogeneity did not act as the primary barrier restricting gene flow (Wang & Bradburd, 2014). In other words, rather than driving the origin of new species, natural selection shapes their local environmental adaptation after primary reproductive isolation. Our findings further highlight that the niches of the species in the *S. sanguinolenta* group do not fully overlap (Figure 5b,c). This suggests that beyond their shared adaptation to cool habitats, these species are subjected to significantly different selection pressures. Consequently, it paves the way for distinct evolutionary trajectories driven by local adaptation, a pattern similarly observed in other taxa, such as Asian seabass (Wang et al., 2016), butterfly bush (Ma et al., 2021). Based on this, we actually need to rely on morphological and genetic evidence to prove that new species are environment‐driven (Albert & Schluter, 2005).

Distinct leaf arrangements across the *Selaginella sanguinolenta* group correspond closely to ecological niche gradients. *S. nummularifolia*, characterized by a fusiform cross‐section, thrives on open, rocky mountain slopes with low temperature and humidity, where solar radiation, elevation, and soil salinity are significantly higher than habitats of other species. In contrast to typical dimorphic spikemosses, which dorsal leaves are relatively small to optimize light capture, *S. nummularifolia* is distinguished by its massive, thickened dorsal leaves that entirely cover the stem and the papery, functionally non‐photosynthetic ventral leaves. This phenomenon is common in alpine plants; for example, the wooly and overlapping leaves of *Eriophyton wallichii* work to enhance water repellency, solar radiation absorption, and the accumulation of leaf heat (Peng et al., 2015). *S. rossii*, *S. jacquemontii*, *S. sajanensis*, and *S. baodongii*, exhibiting a horizontal H‐shaped cross‐section (alternate arrangement of smaller dorsal and larger ventral leaves, portions of the stem exposed), typically inhabit more humid river valleys at lower elevations. Such structural adaptation aligns with ecological patterns wherein leaves in warmer, more humid environments tend to be larger, thinner, and less densely packed (Lin et al., 2017; Lusk et al., 2019). Ventral leaves of *S. jacquemontii* that can be flat or clasping may be influenced by seasonal monsoon rainfall and weak solar radiation. It is essential to consider that natural selection during speciation also reflects paleoenvironmental factors. The “open” leaf arrangement of *S. sajanensis* at higher latitudes may relate to warmer climates of the late Oligocene to Miocene (Mentzer et al., 2025) or adaptations to limited sunlight. Occupying an intermediate ecological and morphological state, *S. aitchisonii* and *S. sanguinolenta* inhabit a transitional zone. Their vegetative leaves clasp the stem to form a subquadrate (rounded‐square) cross‐section, lacking pronounced dorsoventral differentiation. In these species, the dorsal leaves do not fully overlap the ventral leaves, allowing the latter to retain photosynthetic capacity. This intermediate morphology precisely mirrors their habitat where cool, moderately moist, and open environments.

Furthermore, we observed the accumulation of genetic differences between species pairs throughout the whole genome. Tajima's *D* statistic indicates a slight tendency for rare genetic variants to increase throughout the group, and Fay and Wu's *H* consider the absence of a selective sweep signal (Figure 6e,f); these suggest that the *S*. *sanguinolenta* group is experiencing recent population growth or may be under weak positive selection. Further comparison of different species revealed that two widespread species (*S. aitchisonii* and *S. jacquemontii*) showed a more obvious tendency to be selected, while the other five species were more likely to have experienced a decrease in population size and/or balancing selection. The trend of historical changes in effective population size also seems to support the above explanation. Moreover, considering that large populations are not the common in this group, the effect of genetic drift in small populations would be particularly pronounced (Kliman et al., 2008). As a random (chance) mechanism, genetic drift may increase or decrease the frequency of any allele and may therefore sometimes even lead to the loss of beneficial alleles or the fixation of harmful alleles, thus affecting neutral evolution measures, leading to the detection deviation (Schmidt & Pool, 2002).

GO functional enrichment analysis of the highly differentiated regions, as supplementary evidence to explore the potential role of natural selection, revealed significant divergence in biological processes among the species pairs. To ensure statistical rigor, we focused only on terms that remained significant after Benjamini–Hochberg correction (Table S13). While the comprehensive results for all pairwise comparisons are provided in the Supplementary Material, we highlight a few representative examples here. For example, protein modification process (GO:0036211) and macromolecule modification (GO:0043412) were consistently identified across the majority (ca.70%) of species pairs. Previous studies have widely reported the crucial roles of these specific regulatory processes in plant responses to various environmental stresses, such as heat (Ma et al., 2022), drought (Ackah et al., 2024), hypoxia (Zhang et al., 2025), and freezing (Luo et al., 2023). Additionally, specific molecular functions such as hydrolase activity (GO:0016887, GO:0017111, GO:0016818, GO:0016817) were significantly enriched in pairwise *S. rossii* versus *S. sajanensis*. Another specific case, our niche analysis indicated that *S. nummularifolia* occupies significantly harsher habitats—characterized by stronger solar radiation, higher elevations, and greater soil salinity—compared to *S. aitchisonii*. Consistent with this ecological divergence, several GO terms significantly enriched (after BH correction) between this specific species pair, such as secretion (GO:0046903), export from cell (GO:0140352), exocytosis (GO:0006887), and vesicle‐mediated transport (GO:0016192), point towards potential physiological adjustments. While these functional differences may reflect underlying environmental adaptations, additional physiological validation would be required to explicitly link them to specific ecological stressors.

### Population contraction yet survival under paleo‐environment change

Given the ancient divergence of the *Selaginella sanguinolenta* group, the contemporary status of these species is not solely determined by initial speciation events. Reconstructions of historical effective population sizes reveal that, post‐speciation, these species underwent several major events that profoundly affected their population sizes, distributions, and persistence. This demographic history can be divided into three stages. Stage I, Late Miocene–Early Pliocene: This represents the most recent warm period, during which temperatures were 2 to 3°C warmer than pre‐industrial conditions (Mentzer et al., 2025). Throughout this stage, all seven species of *N*_e_ gradually expanded. *S. aitchisonii* first reached its maximum at the Miocene–Pliocene boundary, while the other species followed suit, reaching their peak around the early Pliocene in a global cooling trend. Stage II, Late Pliocene–Early Quaternary: *N*_e_ declined to varying degrees. This decline may be influenced by the anomalous global cooling during Marine Isotope Stage 2 (MIS2, ca. 3.3 mya), a short glaciation event thought to have interrupted a period of global warming (Lisiecki & Raymo, 2005). Notably, *S. aitchisonii* exhibited a transient population expansion prior to this decline, which we hypothesize was linked to localized intensifications of the summer monsoon. Stage III, Pleistocene: Seven species gradually reached a bottleneck amidst the dramatic climate fluctuations and cooling (Crowley & Hyde, 2008) during this period. Then, only four species underwent significant expansions during the Late Pleistocene. As a relatively low‐latitude species, *S. jacquemontii* may have been affected even earlier then *S. nummularifolia*, *S. aitchisonii*, and *S. rossii*. *S. jacquemontii* was the first to initiate demographic recovery, resuming expansion around 0.6–0.4 mya during intermittent warming intervals preceding the Last Interglacial (LIG, ca. 129–116 kya) (Burke et al., 2018).

The SDM results and PSMC estimates generally align, indicating that the historical optimum habitat did not expand significantly even by the end of the LGM (ca. 130 kya). Additionally, a geographically discontinuous distribution pattern emerged during the latest extreme events of the LGM (Heinrich Stadial I, 17.0–14.7 kya). This is not unexpected, as several factors, such as the weakening of the East Asian monsoon, drier conditions in continental regions, the presence of continental ice sheets, and recurring sea ice (Gao et al., 2020), likely contributed to the fragmentation of the distribution of this group. Moreover, as illustrated in Figure 7d, populations exhibiting higher genetic diversity are situated within the optimal habitats identified by SDM, primarily concentrated between 30° N and 45° N (Xinjiang and northwestern China). Previous studies have indicated that the location where plant populations with high genetic diversity can be inferred as potential refuges during climatic changes (Giudicelli et al., 2022; Torre et al., 2022) and may also be beneficial for the recovery of these populations afterward (Zhang et al., 2024). While mountains block genetic flow, they may also serve as natural barriers to the Siberian cold front, such as the Ili River Valley and the Tianshan Mountains. By contrast, genetic diversity is significantly lower in *S. sanguinolenta*, *S. sajanensis*, and *S. baodongii* at higher latitudes. The occurrence of Quaternary glaciations in the Amur region, inferred from relic glacial landforms and moraine deposits (Arzhannikov et al., 2025), likely explains the absence of significant population expansions in these three species.

### Heterospory combining independent gametophyte generations maintain long‐term inbreeding and interspecies isolation

Upon reviewing the evolutionary history of *Selaginella sanguinolenta* group, our findings indicate that these species exhibit genetic patterns which notably highlight the effects of isolation and extreme inbreeding on dispersed populations. We observed high levels of genetic differentiation among species, as well as pronounced divergence within species lineages. Specifically, populations in this group exhibit low genetic variation and a high inbreeding coefficient, which means each species is assigned to a single and unique gene pool under cluster analysis (De Groot et al., 2012). The *F* values for species in *S*. *sanguinolenta* are nearly 1 (Table 1), indicating a tendency towards extreme self‐fertilization rather than a mixture of cross‐ and self‐fertilization (Pelosi & Sessa, 2021).

These highly isolated gene pools contrast sharply with those of homosporous ferns and bryophytes. Previous cases of homosporous plants (e.g., *Sphagnum*, *Athyrium*) have widespread genetic connectivity and minimal genetic structuring (Imwattana et al., 2022; Liang et al., 2022; Vogel et al., 1999). This pattern is largely attributed to their remarkable ability to long‐distance dispersal with atmospheric circulation (Gómez‐Noguez et al., 2026) and establish populations from a single spore (Wolf et al., 2001), which frequently leads to their formation of patchy distributions over vast distances (De Groot et al., 2012). Although existing research indicates that *Selaginella* lacks effective long‐distance active dispersal capabilities, even with spore discharge distances (for both microspore and megaspore) not exceeding 120 cm and maximum height under 80 cm even when utilizing slingshot mechanism (Giorgi et al., 1997; Schneller & Kessler, 2020), passive dispersal via wind and atmospheric circulation remains viable (Chamberlain, 1967; Silva et al., 2025). For example, the proportion of *Selaginella* (heterosporous lycophytes) spores detected in the “spore rain” of some cloud forests is comparable to that of homosporous ferns (Gómez‐Noguez et al., 2017). Counterintuitively, this implies that even if the potential for long‐distance dispersal exists, our species group nevertheless maintains strict genetic isolation, which sharply contrasts with the widespread gene flow typically observed in homosporous lineages. We hypothesize that this pattern likely results from the “synaptospory” dispersal mechanism in *Selaginella*, whereby megaspores and microspores adhere to complementary ornamentation (Wang et al., 2018). In other words, this dispersal method may facilitate the scenario where different sexual spores landing in close proximity for reproduction originate from the same individuals, thereby heavily promoting self‐fertilization. Besides, hybridization may not necessarily represent an advantage for *Selaginella*, for example, very early observations indicated that a large percentage of sterility or malformed spindles are more common in suspected hybrids (Graustein, 1930).

Furthermore, the distinction between *Selaginella* and seed plants (the other major heterosporous lineage) is also pronounced. This is particularly evident when comparing *Selaginella* to wind‐pollinated plants, whose pollen dispersal mechanically resembles spore dispersal. In these wind‐pollinated species, extensive gene flow between relatively distant populations is not a rare occurrence, such as in *Populus lasiocarpa* (Li et al., 2023) and *Myricaria germanica* (Fink et al., 2022). By contrast, the independence of gametophytes may place these organisms at a distinct disadvantage in colonization. While recent studies have argued that independent gametophytes should not be deemed a “weaker” generation (Kessler & Aros‐Mualin, 2024), it is important to recognize their reliance on water. This dependency implies that both megaspores and their germinating gametophytes must be situated in environments conducive to successful germination and fertilization. Research on *Isoetes* species (Kott & Britton, 1982) indicates that germination conditions of megaspores and microspores are different, microspores germinate quicker than megaspores, and there are notable differences in the longevity of male and female gametophytes. Together, these physiological constraints render successful colonization and the establishment of viable populations a formidable and highly stochastic event, not to mention the compounded difficulties of interspecific hybridization.

In short, we propose that the combination of heterospory and independent gametophytes severely exacerbates extreme inbreeding in *Selaginella* following initial geographic isolation, thereby further accelerating the process of allopatric speciation among these lineages.

### Complex evolutionary history and study limitations

The phylogenetic incongruence within the *S. sanguinolenta* group has long been a recognized challenge (Tang et al., 2023; Zhang et al., 2022). While previous studies relying on plastid genes or limited nuclear markers proposed that topological conflicts might stem from plastid genome anomalies, hybridization, or incomplete lineage sorting (ILS) (Zhang, Xiang, & Zhang, 2019, Zhang et al., 2022, 2023), our expanded population‐level nuclear genomic (SNPs) data present a more nuanced picture. In our dataset, each species forms an exceptionally robust monophyletic clade, yet widespread topological inconsistencies among the lineages persist.

We preliminarily rule out atypical nuclear mutation rates or widespread hybridization as the primary drivers of this incongruence. Selaginellaceae has long been considered an evolutionarily conserved group (Qin et al., 2023). They have an extremely small nuclear genome and are the only known group of vascular plants that lacks a shared ancient whole‐genome duplication (WGD) event (Banks et al., 2011; Li et al., 2015). Furthermore, our population genetic structure and gene flow analyses show no evidence of recent hybridization or strong introgression among these species. While fully resolving this phylogenetic conflict is beyond the primary scope of this study, our findings indicate that such topological incongruence is closely associated with incomplete lineage sorting (ILS) resulting from complex historical demographic processes. First, the topological statistics revealed that the frequencies of two minor discordant tree topologies were highly symmetric (roughly a 1:1 ratio, e.g., 14.18% versus 12.83%) (Figure S6b). Under the multi‐species coalescent framework, this symmetrical distribution of discordant topologies is the expected signature of incomplete lineage sorting (ILS) (Pamilo & Nei, 1988). Moreover, the possible ancient hybridization origin of *S*. *sanguinolenta* group (Kang et al., 2024) likely provided the rich ancestral polymorphism necessary as a precondition for such widespread ILS. This demographic complexity is further corroborated by the remarkably flattened distribution of topology weightings across all 15 possible trees in deeper nodes (Figure S6), where even the most frequent topology accounts for a low proportion of the genome. This pattern indicates that the concentrated and chaotic geological events in East Asia may have triggered an atypical rapid radiation within this group (Martin & Van Belleghem, 2017). The short internodal times during this radiation would lead to deep coalescent events, thereby driving the severe phylogenetic incongruence (Simões et al., 2016; Whitfield & Lockhart, 2007). Furthermore, as the topologically complex East Asian landscape rapidly fractured, the ancestral range of *S*. *sanguinolenta* group was forcefully divided into multiple isolated blocks. The resulting physical isolation of these primitive populations would easily passively establish the founder effect wherein new lineages randomly carry only a subset of the genetic diversity present in the original population and genetic drift acted as a strict random sampling mechanism (Barton & Charlesworth, 1984). Because of this powerful stochasticity, some anomalies can still be observed even though the overall populations exhibit a significant isolation‐by‐distance (IBD). For example, genetic differentiation between *S. aitchisonii* and *S. jacquemontii* (*F*_ST_ = 0.54) is slightly higher than between *S. aitchisonii* and *S. baodongii* (*F*_ST_ = 0.48), even though the latter are separated by a greater geographic distance (Figure 1). Consequently, it is possible that the current morphological or genetic characteristics of a species reflect the founder effect rather than the stepped evolutionary order of species (Itescu et al., 2014).

Notably, introgression tests detected potential signatures of gene flow events between certain lineages (e.g., from *S. rossii* to *S. jacquemontii‐*northwest and southwest China lineage, as estimated by TreeMix, Figure 5b). Given our findings of clear interspecific genetic structure, high genetic differentiation, and strict geographical barriers, the possibility of recent genetic exchange among these currently isolated species is rejected. Instead, we propose that these statistical signals may be caused by two possible mechanistically distinct scenarios. For one, these signals could represent analytical artifacts where complex demographic processes confound introgression tests. Unresolved ILS during rapid radiation (Pease & Hahn, 2015), historical population bottlenecks (Figure 7) (Martin et al., 2015), or the genetic influence of unsampled populations or extinct taxa (often referred to as ghost lineages) (Tricou et al., 2022) are all mechanisms capable of generating false‐positive signatures of introgression. For another, they may represent potential real biological evolutionary processes, specifically ancient reticulate evolution that occurred prior to complete geographical isolation. As widely documented in diverse early adaptive radiations and broadly across eukaryotes, such historical admixture leaves residual genomic signatures that persist in descendant lineages long after speciation (Fontaine et al., 2015; Mallet et al., 2016; Pease et al., 2016).

To explicitly test these hypotheses, future studies may pay more attention to phylogenomic analyses that incorporate more sophisticated network models. Moreover, while this study provides a comprehensive framework for the *S. sanguinolenta* group, broadening the research scope to include other diverse lycophyte groups is essential. Comparative studies across these ancient, early‐diverging vascular plants will be crucial to determine whether the unique evolutionary mechanisms (e.g., extreme inbreeding coupled with strictly geography‐driven divergence) we observed would represent general evolutionary rules for heterosporous with independent gametophyte lineages in response to profound geological and climatic shifts.

## MATERIALS AND METHODS

### Sequencing and assembly

The materials of *Selaginella aitchisonii* collected from Xuanhua district, Zhangjiakou City, Hebei Province, China. The total genomic DNA was isolated from fresh plant tissues with the CTAB method as described in Li et al. (2013). One paired‐end (PE) library with an insert size of 150 bp was constructed and sequenced on the Illumina HiSeq 2000 platform (Illumina, San Diego, California, USA). A total of 11.48 Gb of Illumina reads with ~85 × coverage was generated for the genome survey using jellyfish (jellyfish count ‐C ‐m 17 ‐s 1000000000; jellyfish histo ‐h 100 000) and GenomeScope v1.0 (‐m 500 ‐k 17) (Table S1; Marçais & Kingsford, 2011; Liu et al., 2013). For PacBio HiFi library construction, genomic DNA was sheared to 15 Kbp and sequenced on the PacBio Sequel II system by the CCS model. A total of 11.48 Gb of HiFi reads with ~ 58 × coverage was generated (Table S1). Hi‐C library was constructed from cross‐linked chromatins of plant cells using a standard Hi‐C protocol and sequenced on the Illumina NovaSeq 6000. A total of 17.03 Gb of Hi‐C reads with ~126 × coverage was generated. In addition, one PE library with an insert size of 150 bp was performed with the Dynabeads mRNA DIRECT Kit (invitrogen) and sequenced on the Illumina NovaSeq 6000 platform. A total of 18.09 Gb of Illumina reads with ~134 × coverage was generated for the prediction of coding protein.

We assembled HiFi reads using hifiasm v. 0.16.1‐r375 with default parameters (Cheng et al., 2021; Gavrielatos et al., 2021), and the awk command (awk ‘/^S/{print “>”$2;print $3}’ aitchisonii.bp.p_ctg.gfa > aitchisonii.bp.p_ctg.fasta) was used to convert sequence graphs in the GFA to FASTA format. Then, the software purge_haplotigs was used to filter redundancy (Roach et al., 2018). The Hi‐C sequencing data were used to anchor all contigs using Juicer v. 1.5 (Durand, Shamim, et al., 2016), followed by a 3D‐DNA scaffolding pipeline (Dudchenko et al., 2017). Scaffolds were then manually checked and refined with Juicebox v. 1.11.08 (Durand, Robinson, et al., 2016). For sequence consistency assessments, the Illumina short reads and HiFi reads were mapped to the *S. aitchisonii* genome by using a series of software packages, including BWA v0.7.12‐r1039 (Li & Durbin, 2009), minimap v2.1 (Li, 2018), samtools v1.9 (Li et al., 2009). For conservation gene assessments, the BUSCO (−l: embryophyta_odb10, viridiplantae_odb10, respectively) (Seppey et al., 2019; Simao et al., 2015) was used to assess the completeness of the genome assembly. In addition, LTR Assembly Index (LAI, with parameters: ‐window 5 000 000 ‐step 500 000) pipeline was used to assess the accuracy of the genome assembly (Ou et al., 2018).

### Repeat annotation

The identification of transposable elements (TEs) was based on a combination of homology and *de novo* predictions. We first customized a *de novo* repeat library of the genome by RepeatModeler with default parameters (combined with RECON v1.08, RepeatScout, and LtrHarvest) (Bao & Eddy, 2002; Ellinghaus et al., 2008; Flynn et al., 2020; Price et al., 2005). Then, the full‐length long terminal repeat (LTR) and non‐redundant LTR library were identified by LTR_retriever with default parameters (combined with both LTR harvest and LTR_finder) (Ou & Jiang, 2018; Xu & Wang, 2007). A non‐redundant species‐specific TE library was constructed by combining the de novo TE sequence library with the known RepBase (version 19.06) database. Then, the TE sequences in the *S. aitchisonii* genome were initially searched for homology and classified using RepeatMasker v4.1.2‐p1 (Tarailo‐Graovac & Chen, 2009). To annotate tandem repeats in the *S. aitchisonii* genome, we used Tandem Repeats Finder (Benson, 1999) with parameters 2 7 7 80 10 50 500 ‐f ‐d ‐m and converted the result into GFF format using python. Finally, we integrated the above annotation results with the coding sequence by using EDTA v2.1.0 (Ou et al., 2019).

### Gene prediction and annotation

We integrated *de novo* prediction, homology search, and transcript‐based assembly to annotate protein‐coding genes in the *Selaginella aitchisonii* genome. We used StringTie v2.2.1 (Pertea et al., 2015) to predict transcripts, trained using Augustus v3.4.0 (Stanke et al., 2006) with *Arabidopsis thaliana* gene models. The StringTie results were taken as input of GeMoMa v1.9 to polish the prediction. Furthermore, we adopted PASA v2.5.2 (Haas et al., 2003) to predict non‐reference transcriptome based on Trinity v2.14.0 (Grabherr et al., 2013). Additionally, we ran Braker2 (Brůna et al., 2021) to enrich predicted genes, which were supported by transcriptomic data. In combination, gene models obtained from the above approaches were integrated and updated by EVM v1.1.1 (Haas et al., 2008) and structurally updated by PASA v2.5.2 (Haas et al., 2003). The functional annotation of the gene models was performed using BLAST (searching GenBank Non‐Redundant, NR, 20200921), eggNOG‐mapper (‐‐evalue 0.001 ‐‐score 60 ‐‐pident 40 ‐‐query_cover 20 ‐‐subject_cover 20 ‐‐tax_scope auto ‐‐target_orthologs all ‐‐go_evidence non‐electronic ‐‐pfam_realign none), InterProScan (‐goterms), and Kofam_scan v.1.3.0 (with parameters: ‐E 1e‐5) (Aramaki et al., 2020; Cantalapiedra et al., 2021; Huerta‐Cepas et al., 2019; Jones et al., 2014).

### Population sampling and genome resequencing

We collected 191 individuals of seven species from 49 populations in the *Selaginella sanguinolenta* group throughout their main distribution in East Asia (Figure 1, Table S6). Voucher specimens of each population were preserved in Herbarium PE. All micrographs were then photographed by Nikon SMZ 1000 and Leica S9D stereo microscope.

In this study, we conducted whole‐genome resequencing of all samples. Genomic DNA was isolated from Silica gel dried leaf material using Plant Genomic DNA Kit. (DP305, Tiangen Biotech, Beijing, China) with quality checked by agarose gel electrophoresis. The library preparation steps included DNA fragmentation by ultrasonic mechanical methods, DNA fragment purification, end repair, adding “A” to 3′‐end, adapter ligation, appropriate DNA fragment sizes separated by agarose gel electrophoresis, PCR amplification, and quality control. We performed 150 bp paired‐end sequencing on the Illumina NovaSeq 6000 platform with 350 bp insert size (Novogene Bioinformatics Technology, Beijing, China). The filtering of raw reads was accomplished using the FASTQ preprocessor function “fastp” v. 0.22.0 (Chen et al., 2018) with filtering process: (1) remove reads containing adapters, (2) remove reads containing *N* > 10%, (3) remove reads with more than 50% of low‐quality bases (quality value ≤10).

### Variant calling and filtering

The clean reads were aligned onto the *S. aitchisonii* chromosome‐level reference genome using the BWA‐MEM algorithm in BWA v0.7.12 (Li & Durbin, 2009). Mapping coverage and depth of mapped reads were analyzed using PanDepth v2.19 (Yu et al., 2024). SAMtools v1.15.1 (Danecek et al., 2021) software was used to convert SAM files into the BAM format, with sorting and indexing. *HaplotypeCaller* tool of GATK v4.4 was applied to identify genetic variants (single‐nucleotide polymorphisms, SNPs) and generate Genomic Variant Call Format (GVCF) file for each individual. CombineGVCFs and GenotypeGVCFs functions (in GATK) were used to merge GVCF files and annotate variant types. Further rigorous SNPs filtering was performed using SelectVariants (in GATK), VariantFiltration (in GATK), VCFtools v0.1.17 (Danecek et al., 2011) and PLINK v1.9, including but not limited to QualByDepth, MappingQualityRankSum, minor allele frequencies, missing rates, linkage disequilibrium (LD). Considering the different data preferences of following analyses, various filtering parameters were applied and recorded in Table S7.

### Population genetic structure and parameters

The population structure was conducted in ADMIXTURE, using the maximum likelihood method. The number of pre‐defined genetic clusters (*K*) ranged from one to 15, and cross‐validation (CV) error was employed to explore convergence and determine the best *K*. We also conducted principal component analysis (PCA) using PLINK to exhibit the clusters the samples. The AMOVA was performed to estimate genetic variation among and within populations using PGDSpider v3.0.0.0 (Lischer & Excoffier, 2012) and Arlequin v3.5.2.2 software (Excoffier & Lischer, 2010).

Based on the analysis of the population genetic structure, we calculated the genetic diversity and differentiation parameters of five levels: group, clades, species, lineages, and populations. We employed VCFtools v.0.1.17 to calculate observed (*H*_*o*_), expected (*H*_*e*_) heterozygosity, and *F* (inbreeding coefficient). Nucleotide diversity (π) and genetic differentiation (average pairwise *F*_ST_ and *d*_XY_) were calculated using *pixy* v1.2.10.beta2 (Korunes & Samuk, 2021).

### Phylogenetic relationships

We constructed several different methods on filtered SNPs for phylogenetic trees to explore the population and species relationships. Considering the distant relatives might affect the quality of the SNPs dataset, this study tried to define outgroups within the group. Finally, combining previous studies (Zhang et al., 2022, 2023) and our test results based on nuclear rDNA markers (18S, 5.8S, and 26S) (Figure S10), we selected *S. sajanensis* as outgroups for a rooted phylogenetic tree.

First, an unrooted neighbor‐joining (NJ) tree based on Nei's *D*_A_ genetic distance (Nei et al., 1983) in MEGA X software (Sudhir et al., 2018) and 1000 bootstrap replicates. Second, we generated a maximum likelihood (ML) tree by IQ‐TREE2 with the HKY + F + R2 + ASC model identified as the best model using ModelFinder (−m MFP) (Kalyaanamoorthy et al., 2017), and 1000 ultrafast bootstrap replicates were performed (Hoang et al., 2018). Third, SVDQuartets (Chifman & Kubatko, 2014) ran in PAUP4 to estimate the topology of the species tree under the multi‐species‐coalescent model. To investigate group reticulate evolution and the possibility of outcrossing, SplitsTree v4.16.2 was used to construct a phylogenetic network. Additionally, since the phylogenetic topologies within the *S. sanguinolenta* group were reported to be unstable in structure (Kang et al., 2024; Zhang et al., 2022), we further used TWISST software to calculate the proportion of different topological structures; 100 SNPs were used as units, and five‐tips analysis in 15 topologies were estimated.

### Gene flow

We investigated historical genetic introgression using Patterson's *D* (ABBA‐BABA statistic) and the *f*_4_ ratio in using the *Dtrios* program, the *f*‐branch statistic values for each phylogenetic branch using the *Fbranch* program in Dsuite v.0.4 r43; the obtained *f*‐branch statistics were visualized using the dtools.py script. Splits and mixtures among populations were reconstructed using TreeMix v1.13, which utilized allele frequency data from multiple populations to infer a maximum likelihood tree representing their evolutionary relationships, from 0 to 10 migration events (*m*), with 5 replicates of each. The parameter “‐global,” “‐se,” “‐noss” options to calculate the standard errors of the migration proportions and prevent overcorrection. The optimal number of migration edges was determined by maximum ad hoc statistic Δ*m* and 99.8% of variance with R package OptM v.0.1.8 (Fitak, 2021). We also calculated *N*_m_ gene flow values (formulas: *N*_m_ ≈ (1‐*F*_ST_)/4*F*_ST_) as an estimate of gene flow (Slatkin & Barton, 1989) and the relative influence of gene flow versus genetic drift.

### Factors influencing genetic differentiation and natural selection analysis

Effects of isolation‐by‐distance (IBD) and isolation‐by‐environment (IBE) on genetic differentiation were detected by the Mantel test (Spearman) in R package vegan (Dixon, 2003), the program runs with 10 000 permutations. To disentangle the independent effect of environmental heterogeneity from geographic separation (Wang & Bradburd, 2014), we further conducted a partial Mantel test for IBE. We calculated pairwise genetic distance using *F*_ST_/(1‐*F*_ST_) among populations, and pairwise geographic distance matrix was transformed using R package geosphere (Hijmans et al., 2016). The 18 environmental variables were used including temperature, precipitation, soil, solar radiation, wind, and topography (Table S11) were transformed using PCA, and we selected the first three principal components applied Euclidean distance between population pairs as the environmental distance. For evolving neutrally assessment, Tajima's *D* (Tajima, 1989) and Fay and Wu's *H* (Fay & Wu, 2000) were calculated using VCFtools and the script of Pan et al. (2022). Niche overlap statistics Schoener *D* and Warren *I* in ENMTools 1.4.4 (Warren et al., 2010) were calculated to test the level of niche divergence among species. Each environmental variable was further compared by an analysis of variance (ANOVA) with Fisher's LSD.

### Outlier detection and gene functional enrichment

To identify the genomic region with high divergence, we detected the window (10 000 bp) *F*_ST_ with standardized (Z‐score *F*_ST_ = (window *F*_ST_ ‐ mean *F*_ST_)/standard deviation *F*_ST_) between paired species, and the top 2% windows were considered as outliers. We then extracted genes within these detected outlier regions and performed functional enrichment analysis using Gene Ontology (GO) by TBtools (Chen et al., 2023) to determine whether these candidate genes were enriched with any functional classes of genes linking to molecular functions, biological processes, and cellular components. We calculated *P*‐values of Fisher's exact test, and GO terms with an uncorrected *P* < 0.05 were considered significantly enriched, but only those with a BH corrected *P* < 0.05 were further regarded as true biologically relevant signals.

### Demographic history

We followed the snapp_prep.rb script running in BEAST v2.7.4 to estimate divergence time with SNP data. For time calibration, we applied the time “crown” divergence 17.9 mya from Kang et al. (2024), with approximately a lognormal distribution centered at 17.9 mya that has a standard deviation (in real space) of 0.24. To examine the evolutionary histories of the *S. sanguinolenta* group, a PSMC analysis was performed to infer the dynamic changes in the effective population size (*N*_*e*_) over time for each species. To simulate the historical varying distribution of seven species, we also conducted maximum entropy models using MaxEnt v.3.4.1 with current and paleoclimate data (see the information in Table S11). Models with the area under the receiver operating characteristic curve (AUC) (Figure S11) greater than 0.75 were considered reliable. The average layers of 10 replicate runs are divided into four levels of suitable habitats using the natural breakpoint method.

## AUTHOR CONTRIBUTIONS

TY, RW, and XZ designed the research; TY, JY, and NW analyzed the data. TY, JY, NW, RW, and SL discussed the data, and TY, JY, and RW wrote the paper. RW and XZ provided financial and logistic support.

## CONFLICT OF INTEREST

The authors declare no competing financial interests.

## Supporting information


**Figure S1.** CV error of ADMIXTURE analyses.
**Figure S2.** Principal component analysis of genetic structure.
**Figure S3.** Linkage disequilibrium (LD) decay of seven species in *Selaginella sanguinolenta* group.
**Figure S4.** Maximum likelihood (ML) tree inferred by IQ‐TREE2 based on SNPs.
**Figure S5.** Five repetitions of TreeMix results with OptM outputs.
**Figure S6.** Phylogeny relationships of seven species (in two five‐lineages plans) inferred by TWISST topology weighting.
**Figure S7.** *N*_m_ gene flow values (formulas: *N*_m_ ≈ (1‐*F*_ST_)/4*F*_ST_) between species pairs.
**Figure S8.** Leaf arrangement of seven species' young branches in *Selaginella sanguinolenta* group.
**Figure S9.** Counts of candidate genes with highly differentiation windows and GO enrichment terms.
**Figure S10.** Maximum likelihood (ML) tree inferred by IQ‐TREE2 based on rDNA markers.
**Figure S11.** Receiver operating characteristic (ROC) and area under the ROC curve (AUC) over ten replicate runs.

**Table S1.** Sequencing data statistics of *Selaginella aitchisonii* (Unit: Gbp).
**Table S2.** K‐mer statistics of the genomic characteristics of Selaginella aitchisonii obtained by GenomeScope v1.
**Table S3.** Preliminary assembly of *Selaginella aitchisonii*.
**Table S4.** The final assembly of *Selaginella aitchisonii* genome.
**Table S5.** Repeat sequence annotation of *Selaginella aitchisonii* genome.
**Table S6.** Sample information of whole‐genome resequencing in this study. Total of 191 individuals of the *Selaginella sanguinolenta* group.
**Table S7.** Summary of applied SNPs filters for different analyses in this study.
**Table S8.** Summary of SNPs sites after filtering utilized in different analyses.
**Table S9.** Results of molecular variance (AMOVA) analysis.
**Table S10.** Estimates of introgression across different lineage pairs based on *D* statistic and *f*_4_ ratio.
**Table S11.** Environmental variables used in this study.
**Table S12.** Summary of ANOVA analysis (Fisher's LSD; 95% confidence interval) applied to 18 environmental factors across seven species. (Values with the difference letter are significantly different at *P*‐value <0.05).
**Table S13.** Results of GO enrichment between species pairs.

## Data Availability

All whole‐genome resequencing read files are deposited in the NCBI SRA database under the BioProject accession PRJNA1424661. The Hyb‐Seq data and PacBio data of haplotype genome sequencing are under BioProject accession PRJNA1328161: SAMN51699731.
