# Supplementary material for: Population genomics of heterosporous lycophytes: allopatric speciation and historical persistence through paleoclimatic fluctuations
Source: Plant J. 2026 Nov 4;128(3):e71179. doi: 10.1111/tpj.71179 (PMC13735883; doi:10.1111/tpj.71179)
Supplement: Supplementary file 1 [file TPJ-128-0-s002.pdf]

Population genomics of heterosporous lycophytes: allopatric speciation and historical persistence through paleoclimatic fluctuations

TongXin Ye†, Ran Wei†, JiGao Yu, NingXin Wang, SiQi Liang, XianChun Zhang*

Supplementary Figures

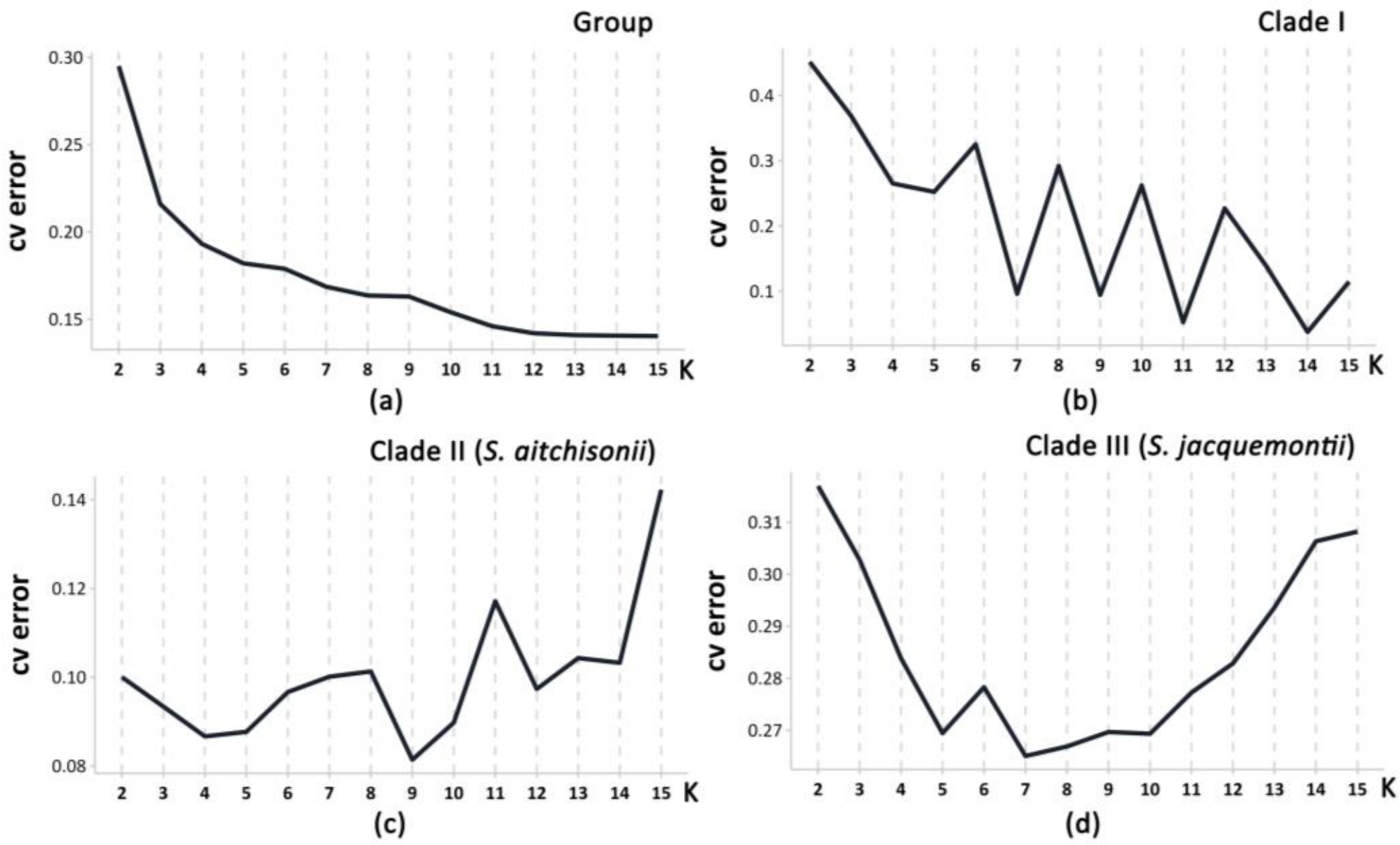

Figure S1. CV error of ADMIXTURE analyses.

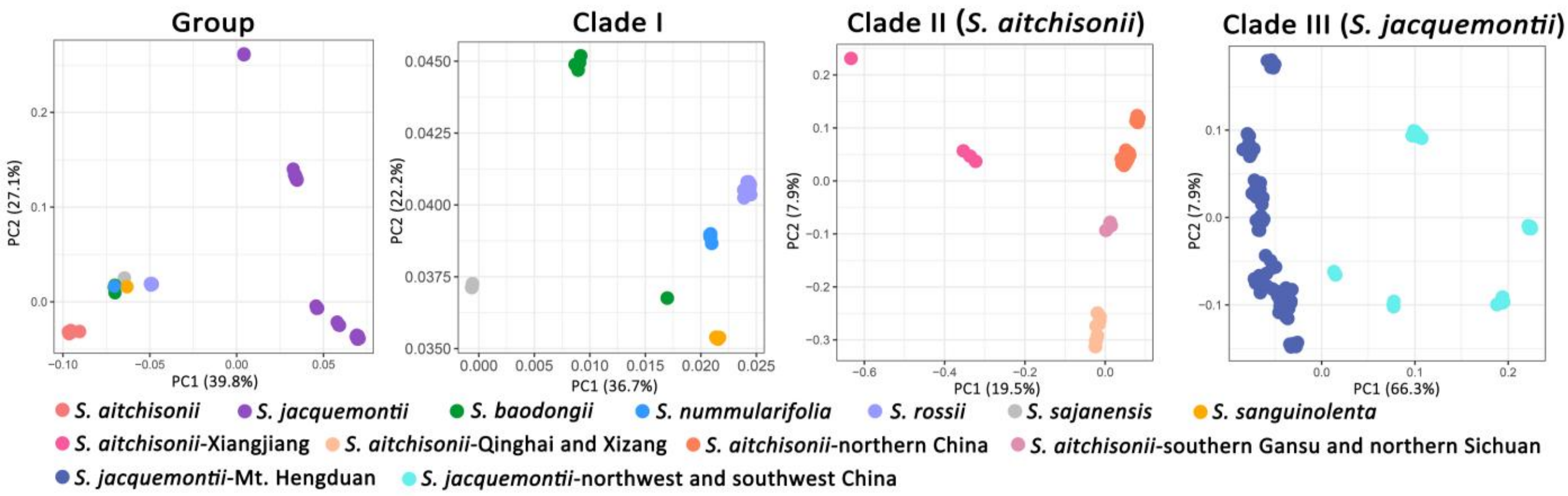

Figure S2. Principal Component Analysis of genetic structure

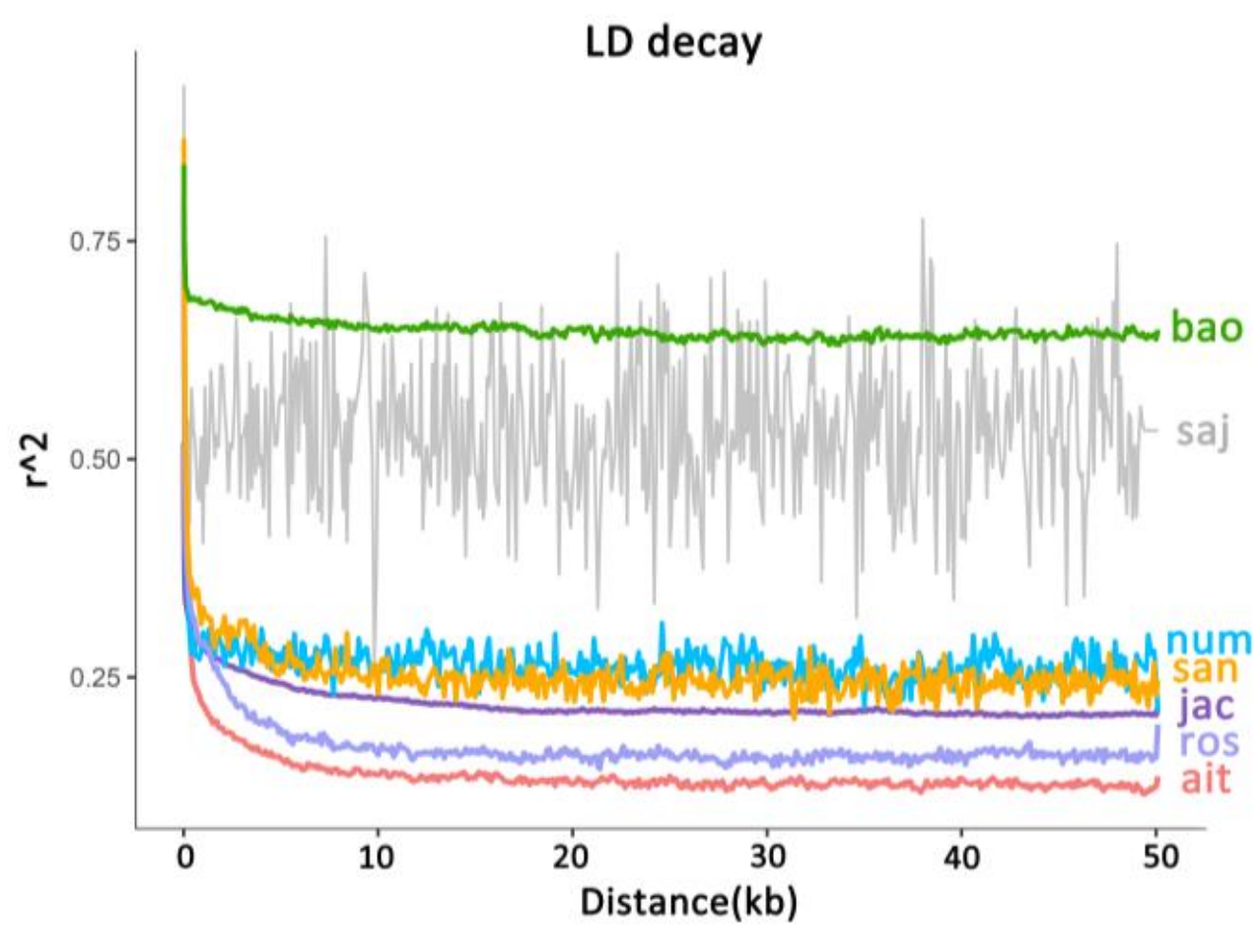

Figure S3. Linkage disequilibrium (LD) decay of seven species in the *Selaginella sanguinolenta* group.

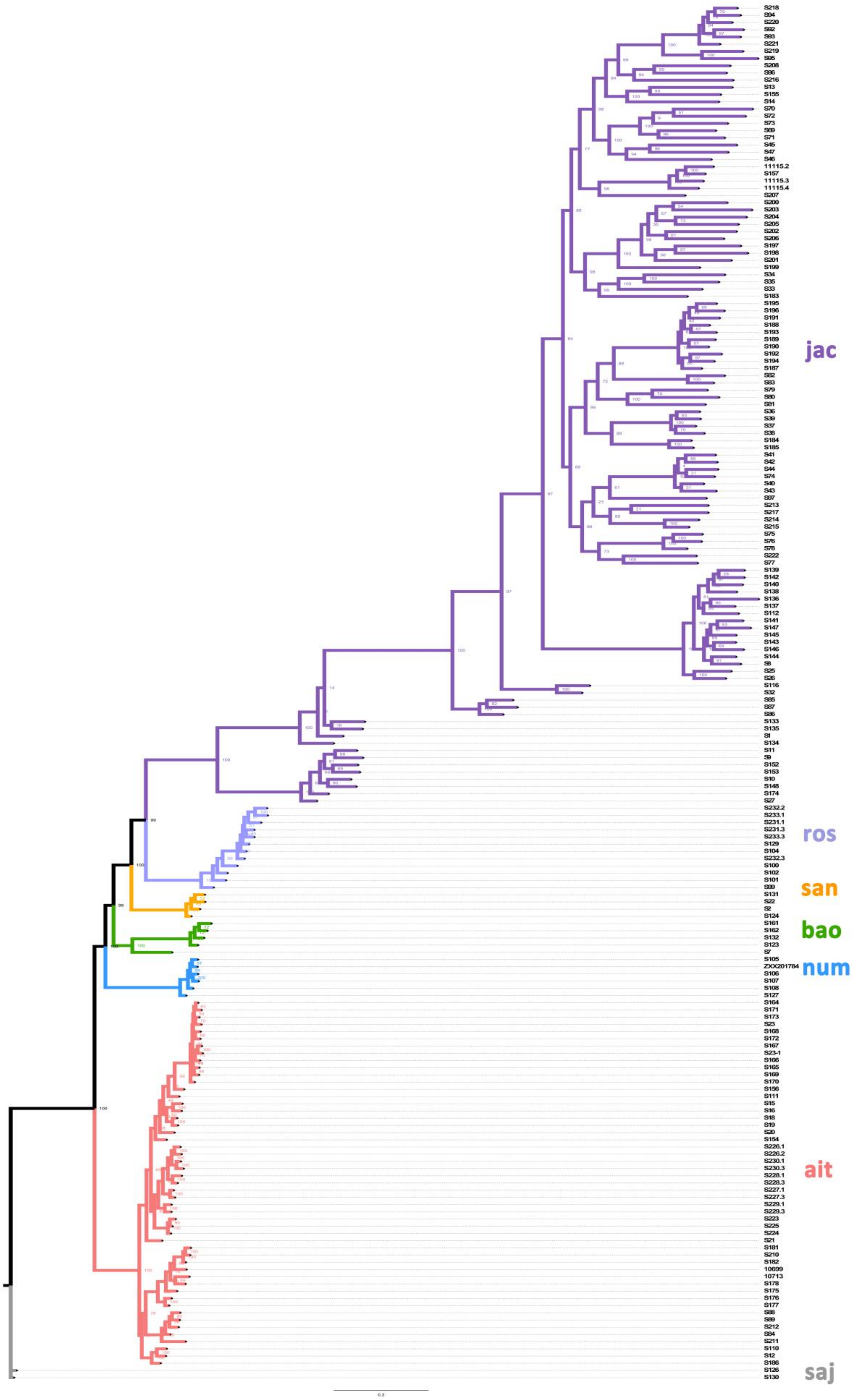

Figure S4. Maximum likelihood (ML) tree inferred by IQ-TREE2 based on SNPs.

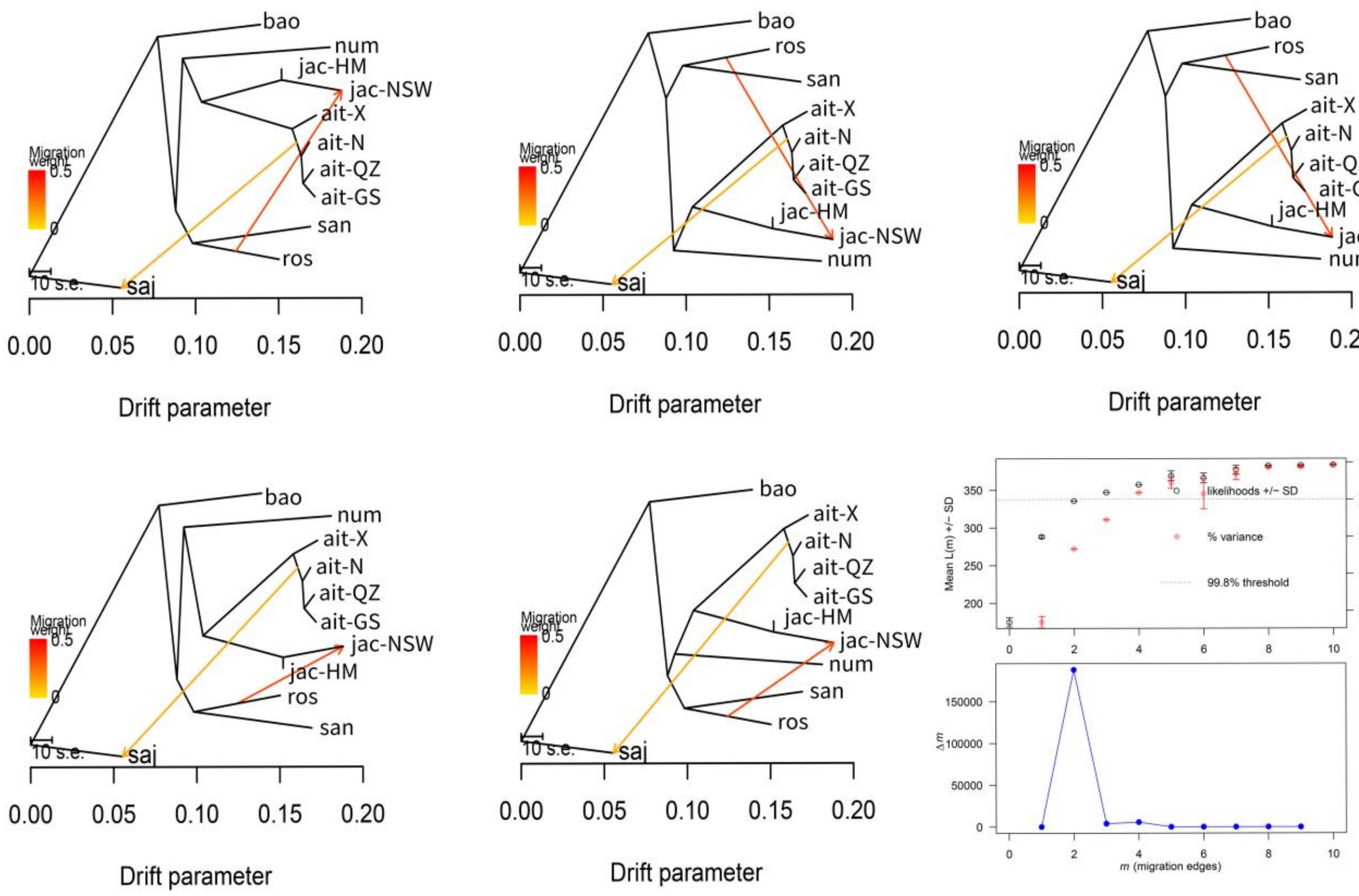

Figure S5. Five repetitions of TreeMix results with OptM outputs.

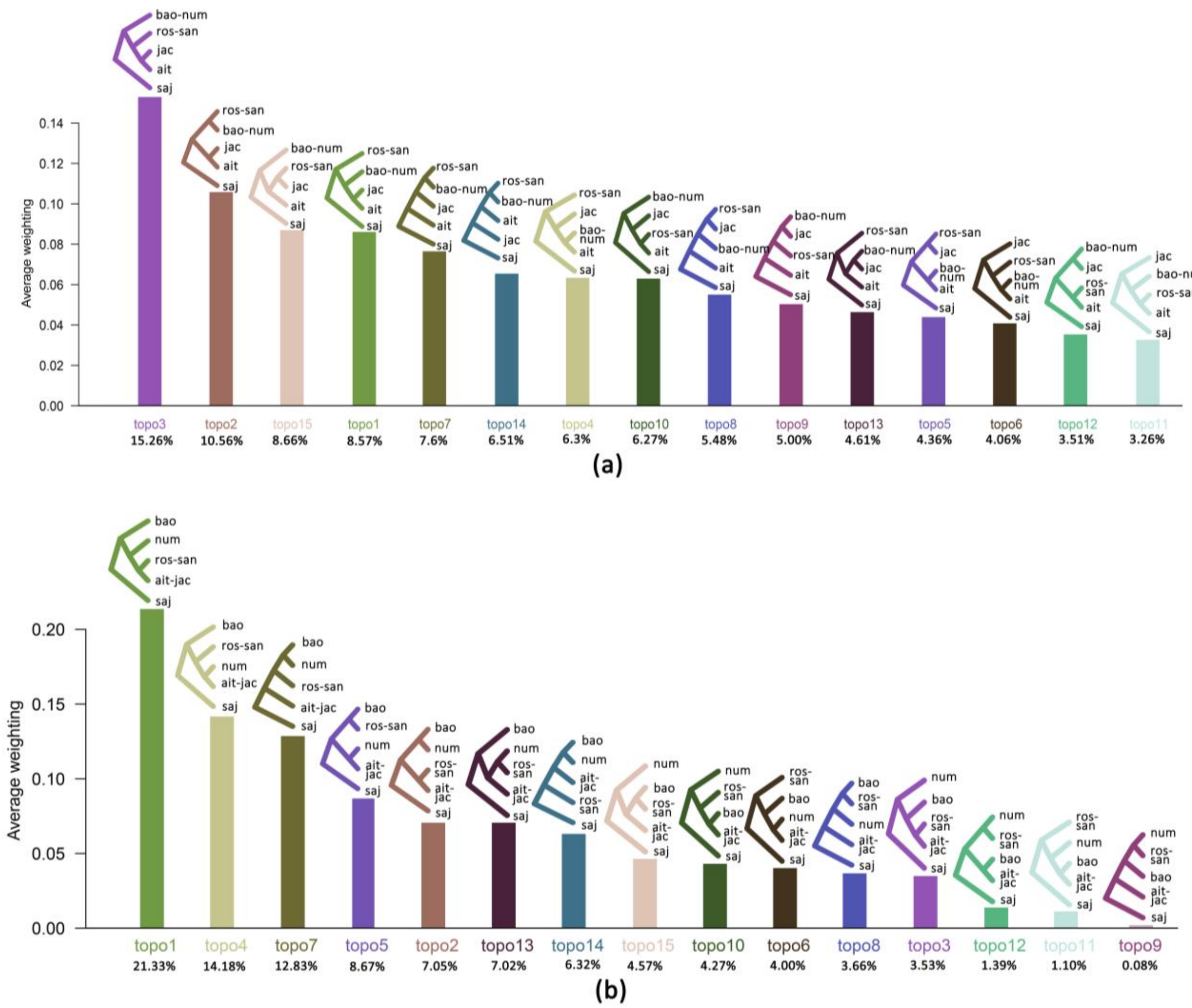

Figure S6. Phylogeny relationships of seven species (in two five-lineages plans) inferred by TWISST topology weighting.

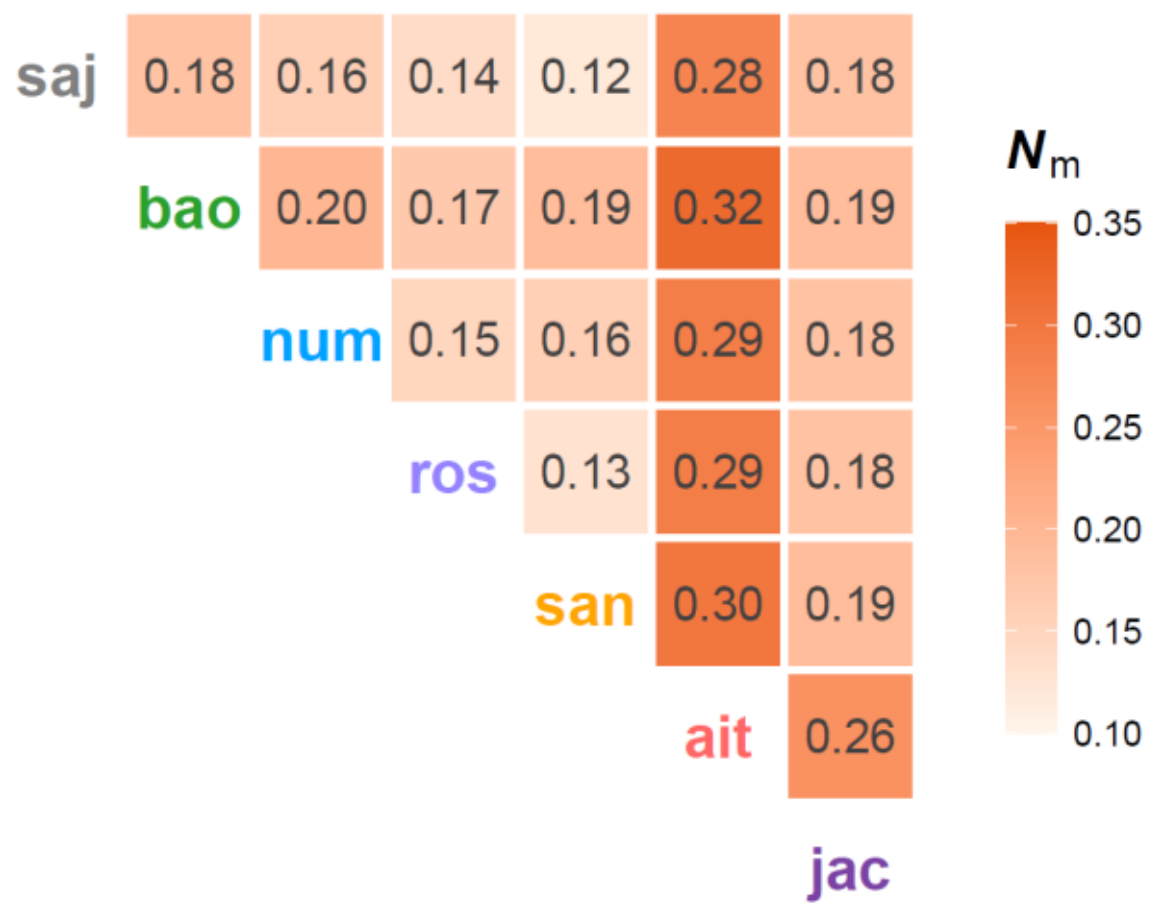

Figure S7. N_m gene flow values (formulas: $N_m \approx (1\text{-}F_{ST})/4F_{ST}$) between species pairs.

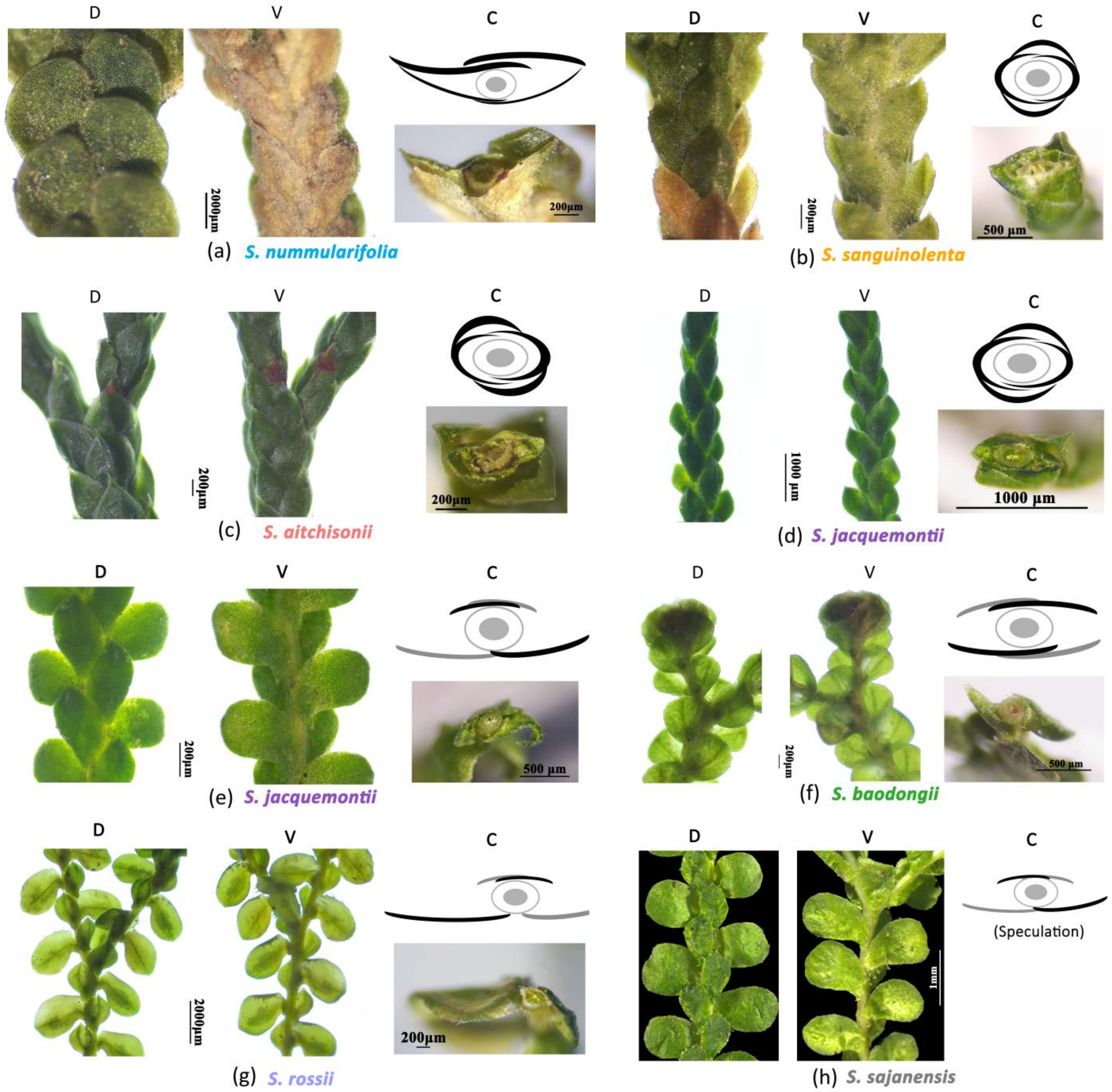

Figure S8. Leaf arrangement of young branches in the *Selaginella sanguinolenta* group in dorsal (D), ventral sides (V) and cross section (C), with three different cross section types: (a) fusiform, (b)-(d) rounded squared, (e)-(h) horizontal H-shape.

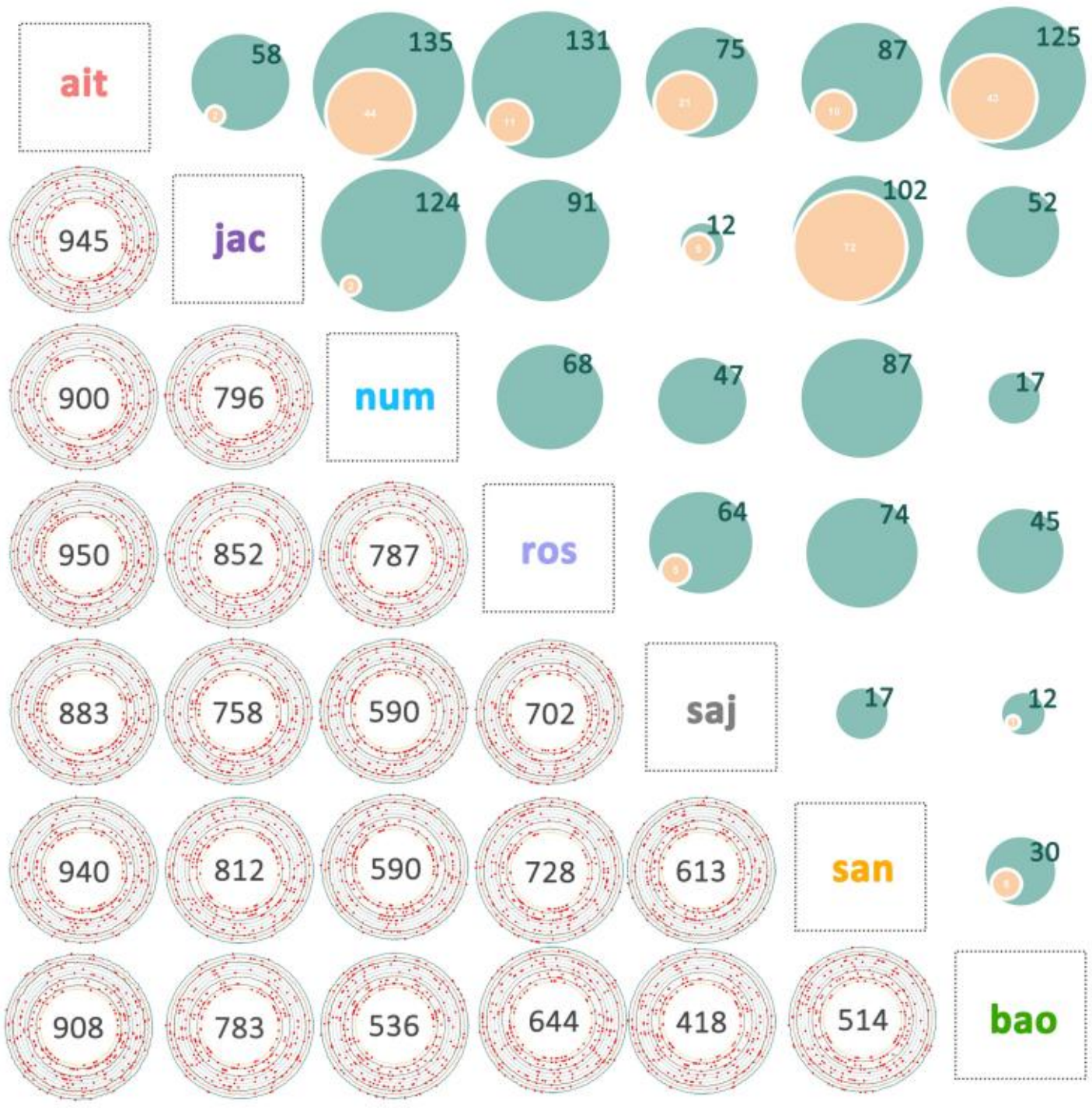

Figure S9. Counts of candidate genes with highly differentiation windows around (lower triangular matrix), and total GO enrichment terms in green circle with the quantity after corrected *p*-value in light orange circle (upper triangular matrix) between species pairs.

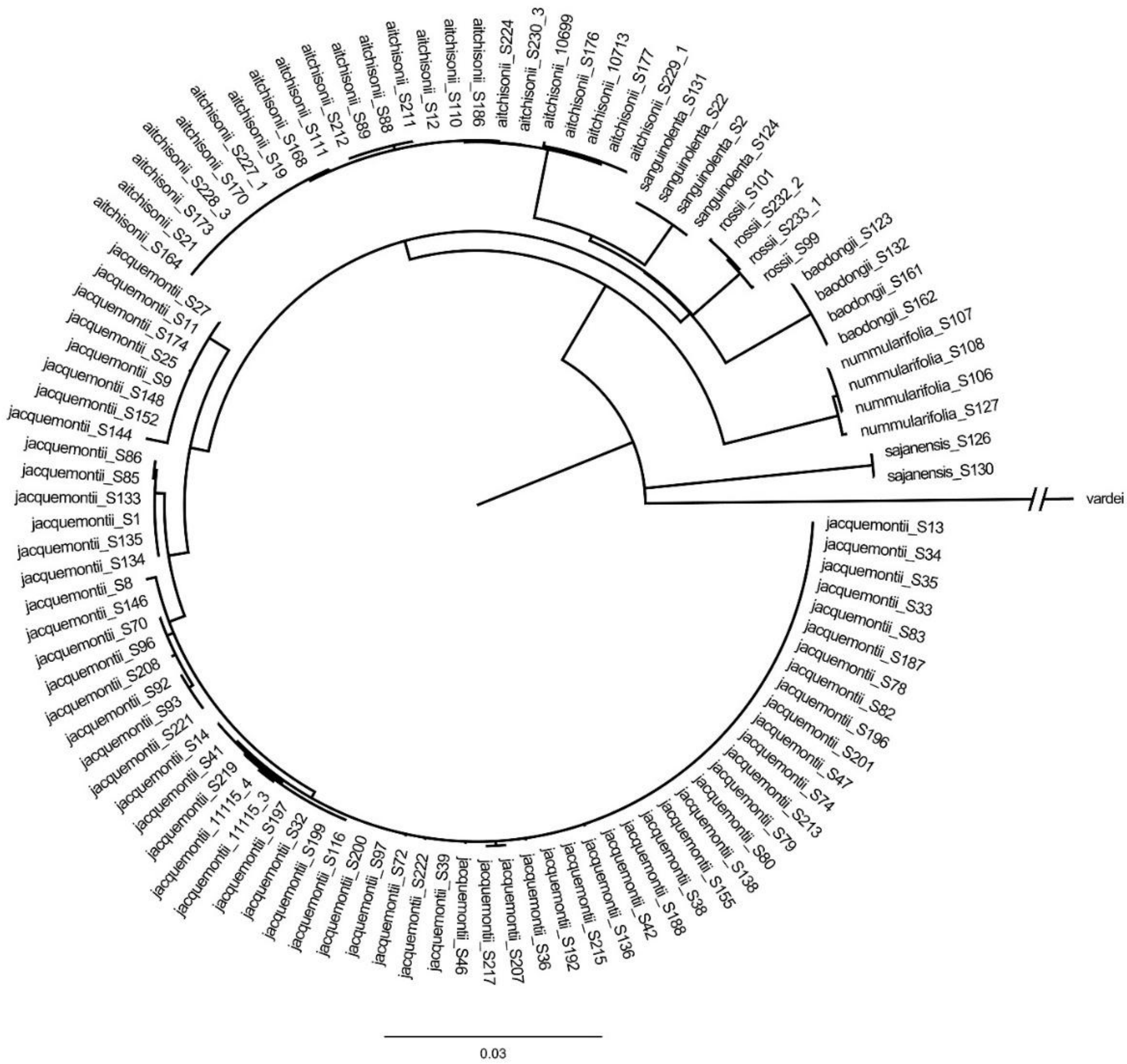

Figure S10. Maximum likelihood (ML) tree inferred by IQ-TREE2 based on rDNA markers. Three rDNA markers (18S, 5.8S, and 26S) and 104 individuals (in *Selaginella sanguinolenta* group) and 1 outgroup (*Selaginella vardei*).

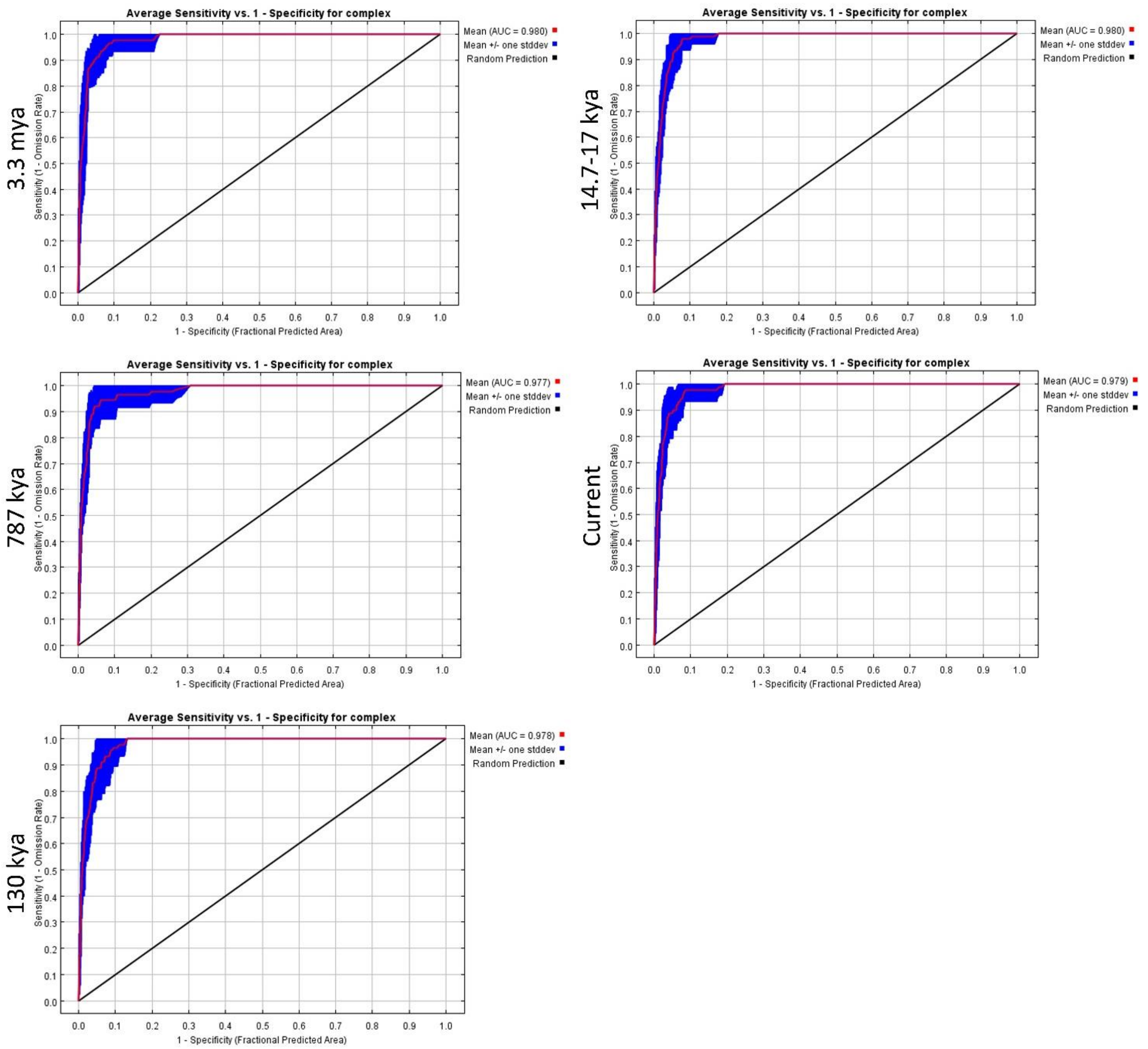

Figure S11. Receiver operating characteristic (ROC) and area under the ROC curve, (AUC) over ten replicate runs
